# NMDA Receptor Antagonist Effects on Speech-Related Mismatch Negativity and Its Underlying Oscillatory and Source Activity in Healthy Humans

**DOI:** 10.3389/fphar.2019.00455

**Published:** 2019-05-08

**Authors:** Sara de la Salle, Dhrasti Shah, Joelle Choueiry, Hayley Bowers, Judy McIntosh, Vadim Ilivitsky, Verner Knott

**Affiliations:** ^1^School of Psychology, University of Ottawa, Ottawa, ON, Canada; ^2^Department of Cellular and Molecular Medicine, University of Ottawa, Ottawa, ON, Canada; ^3^Department of Psychology, University of Guelph, Guelph, ON, Canada; ^4^The Royal’s Institute of Mental Health Research, Ottawa, ON, Canada; ^5^Royal Ottawa Mental Health Centre, Ottawa, ON, Canada

**Keywords:** mismatch negativity, *N*-methyl-D-aspartate (NMDA), ketamine, theta oscillations, cortical current density

## Abstract

**Background:** Previous studies in schizophrenia have consistently shown that deficits in the generation of the auditory mismatch negativity (MMN) – a pre-attentive, event-related potential (ERP) typically elicited by changes to simple sound features – are linked to *N*-methyl-D-aspartate (NMDA) receptor hypofunction. Concomitant with extensive language dysfunction in schizophrenia, patients also exhibit MMN deficits to changes in speech but their relationship to NMDA-mediated neurotransmission is not clear. Accordingly, our study aimed to investigate speech MMNs in healthy humans and their underlying electrophysiological mechanisms in response to NMDA antagonist treatment. We also evaluated the relationship between baseline MMN/electrocortical activity and emergent schizophrenia-like symptoms associated with NMDA receptor blockade.

**Methods:** In a sample of 18 healthy volunteers, a multi-feature Finnish language paradigm incorporating changes in syllables, vowels and consonant stimuli was used to assess the acute effects of the NMDA receptor antagonist ketamine and placebo on the MMN. Further, measures of underlying neural activity, including evoked theta power, theta phase locking and source-localized current density in cortical regions of interest were assessed. Subjective symptoms were assessed with the Clinician Administered Dissociative States Scale (CADSS).

**Results:** Participants exhibited significant ketamine-induced increases in psychosis-like symptoms and depending on temporal or frontal recording region, co-occurred with reductions in MMN generation in response to syllable frequency/intensity, vowel duration, across vowel and consonant deviants. MMN attenuation was associated with decreases in evoked theta power, theta phase locking and diminished current density in auditory and inferior frontal (language-related cortical) regions. Baseline (placebo) MMN and underlying electrophysiological features associated with the processing of changes in syllable intensity correlated with the degree of psychotomimetic response to ketamine.

**Conclusion:** Ketamine-induced impairments in healthy human speech MMNs and their underlying electrocortical mechanisms closely resemble those observed in schizophrenia and support a model of dysfunctional NMDA receptor-mediated neurotransmission of language processing deficits in schizophrenia.

**HIGHLIGHTS:**

- Neural effects of NMDA receptor blockade on speech processing were assessed in a ketamine model.
- Ketamine reduced MMN, theta power, theta phase locking factor and regional cortical current density.
- Psychosis-like symptoms induced by ketamine were related to baseline (placebo) neural measures of speech processing.

## Introduction

This electroencephalographic (EEG)-based research project aimed to advance our neural understanding of language processing by assessing the effects of ketamine-induced *N*-methyl-D-aspartate (NMDA) receptor hypofunction on mismatch negativity (MMN) responses to changes in speech stimuli (i.e., speech deviants). We also investigated stimulus-locked time-frequency and event-related spectral perturbation (ERSP) signatures of ketamine-induced changes in speech MMNs. Although frequently assessed in relation to high frequency (beta and gamma) oscillations (Lee et al., 2018), we specifically focused on the evoked and phase-locking activity of the theta band as viable translational biomarkers for investigating brain dynamics underlying language processing deficits, and their treatment with NMDA receptor antagonists. To examine ketamine effects on speech processing at the oscillatory and network level, we combined ERP and ERSP approaches with a source localization technique, using low-resolution brain electromagnetic tomography (LORETA) to assess MMN-associated current density in auditory and language related regions of interest. Addressing the clinical relevance of this work, as multiple lines of evidence have implicated NMDA receptor dysfunction in schizophrenia pathophysiology including impairments in MMN generation, this study examined the relationship of these neural measures to psychotomimetic symptoms produced by acute ketamine administration. This is the first known human EEG study combining sensor- and source-level approaches to assess the neural substrates of speech deviance detection modified by NMDA receptor antagonist treatment and their association with schizophrenia-like symptomatology.

## Background

### Event-Related Potentials (ERP)

Neurophysiological approaches such as EEG-derived averaged event-related potentials (ERP) are used increasingly as biomarkers for aiding our understanding the neural mechanisms underlying normal and abnormal information processing, and to monitor novel drug therapies for treatment of sensory and cognitive processing deficits (Javitt et al., 2008; Bickel and Javitt, 2009; Javitt, 2015). Within the auditory system, sensory processing is reflected in the generation of the MMN, an ERP reflecting the function of the auditory “echoic” memory system, which maintains brief representations of auditory stimulus features. This automatic change-detection response is elicited when the brain detects an infrequent deviance in a stream of sound stimulation, even in the absence of attention (Näätänen and Alho, 1997; Näätänen et al., 2007; Paavilainen, 2013). The MMN is most commonly recorded in the context of the basic “oddball paradigm” where a physically constant “standard” tone is infrequently replaced by a “deviant” tone (e.g., differing in frequency, duration or intensity). The MMN elicited by the deviance is best observed in the deviant-minus-standard ERP as a frontocentrally distributed negativity, typically peaking at 150–200 ms after the onset of deviant stimulus. As MMN and its abnormality can also be elicited by changes of an abstract nature such as a violation of a multi-stimulus pattern or regularity (Avissar et al., 2018; Salisbury et al., 2018), or that of a complex sequential stimulus rule, the MMN is thought to signal a *prediction-error* based on regularity-violation rather than just stimulus-change (Todd et al., 2013).

### *N*-Methyl-D-Aspartate (NMDA) Receptor Function

*N*-methyl-D-aspartate receptors are widely expressed in sensory and higher cognitive brain regions, and dose-dependent MMN deficits are observed with either systemic or local infusion of NMDA receptor antagonists directly into the auditory cortex (Javitt et al., 1996; Javitt, 2000). Evidence in animal models of the role of NMDA receptor hypofunction, either genetic (Featherstone et al., 2015) or pharmacologically induced, on aberrant MMN generation (Harms, 2016) is paralleled by studies in healthy human volunteers administered acute sub-anesthetic doses of ketamine. Ketamine is a non-competitive NMDA receptor antagonist which reduces and delays MMN generation elicited by frequency and duration tone deviants (Rosburg and Kreitschmann-Andermahr, 2016). MMN generation is not modulated either by dopamine (D1/D2) agonists or by psychotomimetics targeting serotonin (5-HT2H) receptors (Bickel and Javitt, 2009; Kantrowitz and Javitt, 2010). Suggesting both limited dopaminergic involvement and relative specificity of NMDA receptor antagonist effects, MMN is considered to be a simple and useful biomarker of NMDA receptor-type glutamate dysfunction (Avissar and Javitt, 2018).

Mismatch negativity has been increasingly used for studying hierarchical levels of acoustic processing involved in speech and language function. General findings of larger MMNs to speech sounds and words compared with unfamiliar sounds and pseudo-words, respectively, suggests that MMN can serve as a reliable probe of phonological, lexical, semantic, and syntactic processes (Näätänen, 1999; Pulvermuller and Shtyrov, 2006; Shtyrov and Pulvermuller, 2007). Our primary objective was to assess the effects of ketamine-induced NMDA receptor hypofunction on speech MMNs, assessing MMN response to ketamine as a function of 5 speech deviants, including changes in syllable frequency, syllable intensity, vowel duration, as well as consonant change and across-vowel changes. Accurate perception of acoustic features of spoken words (e.g., frequency and intensity) and their temporal attributes (e.g., duration or determining where a work, phrase, and sentence ends) are essential for affective social communication and there is evidence that neural processing of phonological features differs from that of non-linguistic auditory information (Näätänen, 1999; Pulvermuller and Shtyrov, 2006; Shtyrov and Pulvermuller, 2007). As meta-analyses of studies examining MMN response to ketamine have shown equivalent reductions in duration and frequency MMNs, we also expect that ketamine will impair MMN generation across deviant types.

### ERO Time-Frequency Analysis

A mechanistic understanding of the effects of NMDA receptor function on MMN requires an evaluation of its impact on event-related spectral oscillatory (ERO) activity across different frequency bands [delta (<4 Hz), theta (4–8 Hz), alpha (8–12 Hz), beta (12–30 Hz), gamma (30–80 Hz)]. EROs can be obtained at levels ranging from single cell to focal field potentials in animals, to large-scale synchronized activities measured at the human scalp (Moran and Hong, 2011). Power in the theta band is particularly prominent during the processing of tone and speech deviants, with studies showing that neural generation of the MMN is accompanied by theta power modulation and theta phase alignment (Fuentemilla et al., 2008; Hsiao et al., 2009; Bishop and Hardiman, 2010; Ko et al., 2012; Choi et al., 2013; Hermann et al., 2014; Koerrner et al., 2016; Corcoran et al., 2018). NMDA receptor antagonists modulate background spontaneous theta oscillations (Lazarewicz et al., 2009; Hunt and Kasicki, 2013) and reduce both MMN and the theta response to auditory deviants in rodents (Lee et al., 2018). Thus, evoked theta activity is proposed as a viable translational biomarker for investigating brain dynamics underlying auditory processing deficits and their treatment with NMDA receptor antagonists. In addition to measuring the power of oscillations associated with the averaged time-domain ERPs, sensitive ‘time-frequency’ analyses of trial-by-trial ERSPs are being increasingly used to provide a more detailed picture of brain dynamics underlying ERPs, which is not available with the averaged time-domain ERP, as seen with the MMN waveform (Lee et al., 2017). One of the most frequently used ERSP measures, which is computed on a trial-by-trial basis and is known to independently (vs. power) affect the amplitude of the averaged ERP, is the phase locking factor [PLF; also known as phase locking index (PLI), or inter-trial phase coherence (ITC)]. This measures the degree of phase consistency across trials, and is commonly interpreted to reflect phase resetting of ongoing oscillations. The frontal MMN has been found to be associated with an increase in theta power for deviant stimuli as well as by theta phase alignment, whereas the temporal MMN has been found to be associated with theta phase resetting with no power modulation (Fuentemilla et al., 2008). This study investigated the time-frequency signature of ketamine-induced changes in speech MMNs with a specific focus on evoked theta power and theta phase locking.

### ERP Source Activity

The neural characteristics of the MMN (amplitude, power, and phase resetting) are presumed to result from activity of neural networks involved in its generation (Fuentemilla et al., 2008). Studies employing dynamic causal modeling, source localization, functional magnetic resonance imaging (fMRI) and positron emission tomography (PET) have shown the MMN to be generated by neural activity and connectivity within a hierarchically organized cortical network involving temporal (bilateral auditory cortex) and predominantly right frontal brain regions (Näätänen and Alho, 1997; Näätänen et al., 2007). In addition to primary (pAC) and secondary (sAC) auditory cortices in the temporal lobe, the inferior frontal gyrus (IFG) is the most studied non-auditory cortical contributor to MMN generation and appears to serve as an evaluator of stimulus relevance (Todd et al., 2012). In the oddball paradigm, its activity may reflect the importance of the violated expectation (prediction error) detected by auditory cortical areas (Todd et al., 2012). Activity within and between cortical areas is essential for auditory perception and oscillatory activity plays a cardinal role in the recruitment of brain regions and their coupling (Lee et al., 2017). Neurons within the IFG, such as those located in Broca’s language area and Broca’s right homolog, are characterized by a well-described theta frequency synchronization with the auditory cortex (Hsiao et al., 2009; Choi et al., 2013). In order to examine ketamine effects on speech processing at the sensory and network levels, we combined ERP and time-frequency/ERSP approaches with a source localization technique, using low-resolution brain electromagnetic tomography (LORETA), a 3D inverse solution, to assess intracerebral current density as a measure of activation of regions of interest (ROI: pAC, sAC, and IFG) during MMN generation. With ERPs and their underlying spectrally analyzed activity, we expected to see ketamine-induced reductions in MMN amplitude, evoked theta power and theta phase resetting (phase-locking), while with source analysis, we predicted reduced current density (activation) in temporal and frontal cortical areas.

### Relevance for Schizophrenia

For a number of reasons, this line of research has potential clinical implications for schizophrenia cognition (Green, 2006; Javitt, 2009a,b; Leitman et al., 2010; Javitt and Freedman, 2015; Javitt and Sweet, 2015) and particularly with respect to improving our understanding of low-level acoustic processing involved in speech and language functions that are distinctively impaired in schizophrenia (Revheim et al., 2014; Brown and Kuperburg, 2015). Among these reasons are: a vast neuroimaging, molecular, and genetic literature supporting a glutamate hypothesis of schizophrenia involving NMDA receptor hypofunction (Carlsson et al., 1999; Coyle et al., 2010; Javitt et al., 2012; Moghaddam and Javitt, 2012; Moghaddam and Krystal, 2012); pharmacological modeling of schizophrenia features, including clinical, cognitive, and sensory deficit symptoms in rodents/healthy humans with acute sub-anesthetic doses of the NMDA receptor antagonist ketamine (Pauvermann et al., 2017; Thomas et al., 2017); findings in schizophrenia of reliable and robust attenuation of the MMN (effect size *d* ∼ 1.00), which is not affected by antipsychotics (Näätänen and Kakkonen, 2009), is more apparent with speech MMNs (Kasai et al., 2002; Fisher et al., 2008) and is associated with reductions in frontotemporal volume/function (Kasai et al., 2002; Kasai, 2004; Yamasue et al., 2004) and diminished theta responses to auditory deviants (Rodionov et al., 2009; Hong et al., 2012; Kaser et al., 2013; Kirino, 2017; Lee et al., 2017). As reduced MMNs predict conversion to psychosis in clinical high risk and reflect a vulnerability to disease progression (Näätänen et al., 2015), and as smaller MMNs to tone deviants are associated with greater vulnerability to NMDA receptor system disruption with ketamine in healthy humans (i.e., they experience greater psychosis-like symptoms; Umbricht et al., 2002) a secondary objective of this study is to examine the relationship between baseline (placebo) frontal speech MMN/oscillatory and source activity and subjective psychotomimetic response to ketamine.

## Materials and Methods

### Experimental Subjects

Twenty healthy, medication-free, non-smoking, right-handed males (age = 20.94, *SD* = 2.44) were recruited through city/university newspaper advertisements. Only males were recruited in order to avoid potential confounding effects of hormonal variations associated with the menstrual cycle in females. Only non-smokers were sampled in order to reduce any potential confounding effects of nicotine and/or nicotine withdrawal symptoms on ketamine response. Following data processing, two of the participants exhibited marked artifact contamination in their EEGs and the final study sample was limited to *N* = 18. Participants were initially screened by telephone for non-smoker status (defined as not smoking a lifetime total of >100 cigarettes, with no smoking in the past 12 months), absence of medical illnesses, as well as psychiatric and alcohol/drug dependence disorders. They were then assessed for psychopathology and personal/family psychiatric history during an in-person screening session using the Structured Clinical Interview (SCID-non-patient version; Williams et al., 1992) for DSM-IV and the Family Interview for Genetic Studies (FIGS; Maxwell, 1992). They also underwent a full medical examination (including ECG), laboratory blood testing (to rule out any significant medical conditions), urine toxicology (to screen for psychoactive substances), an expired air carbon monoxide test to confirm non-smoker status [<3 parts per million (ppm)], and auditory threshold testing to ensure normal hearing.

Any participants with a current/past or family history of an axis 1–2 DSM-IV disorder, abnormal blood/ECG results, positive drug screen (for barbiturates, ketamine, cocaine, benzodiazepines, ethanol, cannabinoids, and opioids), significant medical illnesses [including seizures and recent (<6 months) head trauma with loss of consciousness], or audiometric assessed indication of abnormal hearing (hearing threshold above 30 dB SPL for 1,000 Hz pure tone) were excluded. The study was limited to males to avoid possible variations related to menstrual cycle. All participants provided written informed consent during the in-person screening session and were compensated $75 CAD per test session. This study was approved by the Research Ethics Board of the Royal Ottawa Health Care Group and was conducted in accordance with the Tri-Council Policy Statement on Ethical Conduct for Research Involving Humans.

### Study Design

Participants were assessed within a randomized, double-blind design consisting of two test sessions separated by a minimum of 5 days. Half received the placebo (saline) in their first test session and ketamine in their second test session, while the other half received the treatments in the reverse order.

### Drug Administration

A racemic ketamine or NaCl 0.90% w/v bolus dose was administered over 10 min (0.26 mg/kg), followed immediately by a constant infusion lasting 60 min (0.65 mg/kg), using an Imed Gemini PC-1 pump. The ketamine dose is consistent with previous studies (Krystal et al., 1999; Morgan et al., 2004; Lazarewicz et al., 2009; Kaser et al., 2013).

### Study Procedures

Test sessions occurred in the morning (beginning at 8:00 a.m.), following overnight abstinence from caffeine, food, drinks (except for water), and alcohol. Sessions began with the insertion of an antecubital intravenous line, attachment of a 2-lead ECG (for continuous safety monitoring), and electrode positioning, followed by the administration of the bolus dose and constant infusion. After a 10 min stabilization period (i.e., 10 min into the infusion), EEG recording and auditory MMN paradigm administration began. This was followed by an evaluation with the Clinician Administered Dissociative States Scale (CADSS; Bremner et al., 1998), which is composed of 19 self-report items (0 = not at all; 1 = slightly; 2 = moderately; 3 = considerably; 4 = extremely) and yields three subjective sub-scale scores (amnesia, depersonalization, and derealization) as well as 8 clinician rated items which yields an “observer” sub-scale score. Vital signs and adverse events were analyzed for safety purposes only. Participants remained in the lab until all symptoms had ceased and were not allowed to drive vehicles to or from the test sessions.

### MMN Paradigm

During MMN paradigm administration, participants viewed a silent video (The Blue Planet by British Broadcasting Corporation et al., 2002) while stimuli were presented binaurally through noise-canceling headphones. A fast multi-feature speech paradigm (Pakarinen et al., 2009) was employed to acquire MMNs to phonetic and acoustic changes in speech stimuli. Compared to standard oddball paradigms, which allow for assessment of cortical discrimination of 1–2 sounds features, multi-feature MMN paradigms allow for very fast assessment of extensive auditory discrimination profiles, evaluating central auditory discrimination of multiple auditory attributes (Näätänen et al., 2004). The stimuli consisted of semi-synthetic Finnish-language consonant-vowel (CV) syllables. The standard stimuli were /te:/ and /pi:/ (frequency = 101 Hz, intensity = 70 dB, syllable duration = 170 ms). The deviant stimuli differed from the standards either in syllable FREQUENCY (FO ±8%; 93/109 Hz), syllable INTENSITY (±6 dB), VOWEL-DURATION (-70 ms; 100 ms /te/ and /pi/), CONSONANT (/pe:/ and /ti:/), or VOWEL (/ti:/ and /pe:/). Additional details regarding the creation of these stimuli can be found in Pakarinen et al. (2009). The presentation of these stimuli followed the same sequence as previously outlined (Näätänen et al., 2004): every other syllable was a standard (*P* = 0.5) and every other one of the 5 deviant syllables (*P* = 0.1 each). There were four 5-min sequences including 465 syllables, of which the first 5 were always standards. In two of the sequences the standard syllable was /te:/ and the deviants were FREQUENCY /te:/, INTENSITY /te:/, VOWEL DURATION /te/, CONSONANT /pe:/, and VOWEL /ti:/, whereas in the other two sequences the standard was /pi:/, and the deviants were FREQUENCY /pi:/, INTENSITY /pi:/, VOWEL DURATION /pi/, CONSTONANT /ti:/, and VOWEL /pe:/. The occurrence of the deviants were pseudo-randomized in a way that all 5 deviants appeared once in an array of 10 successive stimuli and the same deviant was never repeated after the standard following it. The order of the two sequences was randomized between participants. Each deviant type was presented 184 times, the stimulus-onset asynchrony (SOA, onset to onset) was 650 ms, and the total recording time was 20 min. Although use of a native (English) speech paradigm may have been preferable with our English speaking sample, this was the only published ‘optimal’ paradigm that allowed for quick, efficient assessment of multiple speech deviant types in a single recording when we initiated our study. The five speech deviations are generally relevant features in speech sounds across most, if not all, spoken languages. The use of a non-native paradigm to derive automatic cortical discrimination profiles has the advantage of reducing the potential confounding influence of higher-order semantic processes (on MMN generation), which are known to interact with emotional processes that are markedly impaired in schizophrenia as reflected by MMN-indexed auditory emotion recognition deficits (Kantrowitz et al., 2015).

### ERP Recording

Electroencephalographic activity was recorded using current pharmaco-EEG standards (Knott, 2000; Saletu et al., 2006; Jobert et al., 2012). This involved the use of a cap (EasyCap, Herrsching-Breitbrunn, Germany) embedded with 30 Ag^+^/Ag^+^Cl^-^ electrodes, left and right mastoids (TP9 and TP10), as well as 2 bipolar electrodes placed on the supra- and sub-orbital ridge and external canthi (to measure vertical and horizontal electro-oculographic activity). An electrode on the nose served as the reference and a ground electrode was positioned at AFz. Electrical recordings were carried out using a Brain Vision QuickAmp^®^ (Brain Products GmbH, Munich, Germany) amplifier and Brain Vision Recorder^®^ (Brain Products GmbH, Munich, Germany) software. Electrical activity was sampled at 500 Hz, with amplifier bandpass filters set at 0.1–100.0 Hz. Electrode impedances were <5 kΩ.

### MMN Processing

**FIGURE 1:**
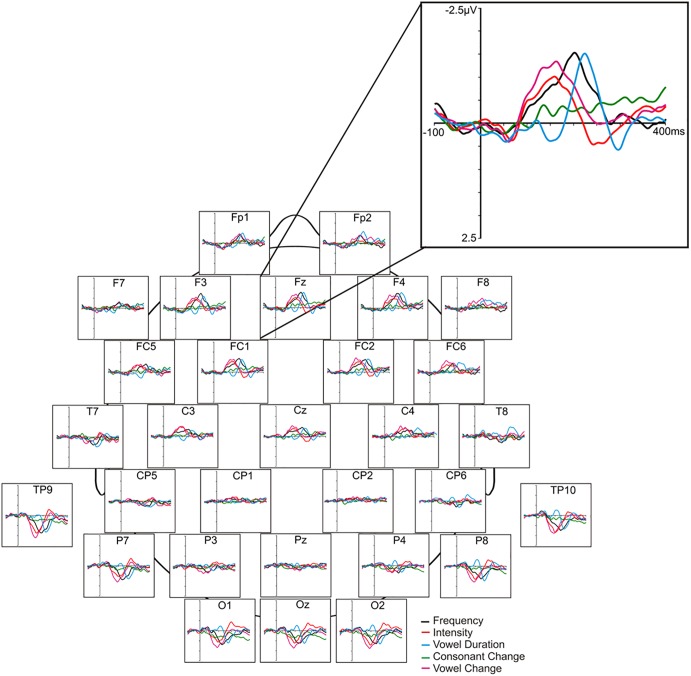
Grand averaged ERP difference waveforms elicited in response to 5 speech deviants in the placebo condition.

Mismatch negativity analysis was completed using Brain Vision^®^ Analyzer 2 software (Brain Products, Munich, Germany). Offline pre-processing involved a visual inspection of the recordings for contamination due to noticeable ocular/muscle/cardiac activity and/or drowsiness, digital filtering at 1–20 Hz, ocular correction with an algorithm (Gratton et al., 1983), segmentation (-100 to 400 ms), semi-automatic artifact rejection (±100 μV), and baseline correction (relative to the pre-stimulus segment). The remaining epochs (minimum 100 per deviant) were then averaged based on stimulus type, and subtraction waveforms were computed (deviant – standard) by digital point-by-point subtraction of standard waveform voltage values from each deviant waveform values. For the purposes of our study and based on grand average waveforms, frontal MMN at Fz, F3 and F4 scalp sites was quantified in terms of peak amplitude (maximum negative voltage relative to average pre-stimulus voltage within the 120–250 ms window for frequency, intensity, duration, vowel, deviants and the 150–280 ms window for consonant deviants). MMN latency (relative to stimulus onset) for each of the deviants, was quantified only at the mid-frontal (Fz) site. The mean number of epochs (±S.E.) for MMN averages was not significantly different between deviants, nor were there differences in epoch numbers across placebo (frequency = 172.67 ± 2.55; intensity = 171.72 ± 2.57; consonant = 170.78 ± 3.02; vowel duration = 171.22 ± 2.68; across vowel = 171.33 ± 2.78) and ketamine conditions (frequency = 174.00 ± 1.77; intensity = 174.78 ± 1.85; consonant = 173.89 ± 1.95; vowel duration = 173.83 ± 1.99; across vowel = 174.72 ± 1.65). In contrast to the original study employing the fast multi-feature Finnish speech paradigm (Näätänen et al., 2004), which employed a mastoid reference and thus evidenced no MMN generation at temporal/posterior sites, our use of a nose reference yielded prominent polarity-inverted MMN responses in these regions, which results from the dipole generator orienting between the auditory and frontal cortices. As a secondary analysis, MMNs were also measured and analyzed at left (TP9) and right (TP10) mastoid sites. Amplitude of the obligatory N1 component (peak negativity between 90 and 120 ms) elicited by the standard stimulus was also measured in order to clarify whether or not ketamine effects were specific to deviance processing vs. general sensory processing. Figure 1 displays the placebo grand averaged difference waveforms [including the polarity reversed MMNs and mastoid (TP9, TP10) sites] for the five speech deviants, together with the grand averaged topographic maps showing amplitude distribution of MMNs across recording sites.

### ERO and ERSP Processing

For each deviant type, the average frontal ERO was obtained for the theta band at Fz, F3, and F4 sites in a manner similar to that of Hall et al. (2011). Time-varying spectral activity (μV) was computed on averaged epochs (separately for each deviant) using a complex Morlet wavelet with a constant [c = σ_f_/f, where σ_f_ = the standard deviation of the centre frequency (f)] value of 5; this was computed over the range of 1–20 Hz with 40 frequency steps. Evoked theta activity (3–7 Hz) within time-frequency ROIs were exported for analysis (±50 ms around peak MMN amplitude). ERO values were then ln-transformed to ensure that the data were normally distributed for statistical analysis. Using the same methodology, theta ERO (within the MMN latency range) was quantified for the standard stimulus to determine whether ketamine effects were specific to deviance processing or generalized to all sensory processing. As background, ongoing oscillations may moderate stimulus-induced oscillatory response, an additional analysis was conducted to assess potential changes in spontaneous theta with ketamine administration. Pre-stimulus epochs of 100 ms duration were digitally sampled (500 Hz) across standard and deviant stimuli and submitted to Fast Fourier Transform (FFT) analysis for computation of ln-transformed spectral amplitude in the theta (3–7) band in the MMN latency range. Values across epochs were summed for statistical analysis.

Phase-locking factor (PLF) values were computed by first performing wavelet transformation on each epoch, and subtracting the standard epochs from the deviants. By using the BrainVision Analyzer “Phase Locking Factor” solution, the average time-frequency PLF values can be extracted. PLF was calculated as 1 minus the circular variance of phases, as described by Tallon-Baudry et al. (1996), and ranges from 0 (random distribution of phases) to 1 (perfect phase locking). Similarly to the ERO data, theta PLF (3–7 Hz) within time-frequency regions of interest was exported for analysis (±50 ms around peak MMN amplitude). PLF was also quantified for the standard stimulus and for spontaneous (pre-stimulus) activity.

### Brodmann Area Regions of Interest

Intracortical current density (A/m^2^) measured at peak MMN activity (based on ERP grand averages) from predefined ROIs were computed using exact low resolution electromagnetic tomography (eLORETA, version 2081104; Pascual-Marqui, 2007; Pascual-Marqui et al., 2011). eLORETA models the cortex as a collection of voxels (6239 voxels with a spatial resolution of 7 mm). Relying on the digitized Talairach atlas and the average MRI brain provided by the Montreal Neurological Institute (MNI) and a cortically restrained solution space, it calculates the non-unique “inverse” problem by computing a three dimensional distribution of intracortical source activity (with zero location error) at each voxel based on surface-level electrical signals. The original LORETA method has received considerable validation from studies using more established localization methods such as structural and functional MRI, PET, and implanted electrode recordings. The selected ROIs were based on eLORETA-defined Brodmann Areas (BA), and current density data from a single centroid representative voxel of each BA (the voxel closest to the center of the BA mass, which is an excellent representation of the corresponding BA) were extracted for further analysis. This included the primary (BA 41) and secondary (BA 42) auditory cortices and the three regions comprising the IFG (BAs 44, 45, and 47). Current density was derived for the left and right hemispheres of each BA.

### Statistical Analyses

Statistical analysis was conducted using SPSS version 23 (SPSS Inc., Chicago, IL, United States). Separate repeated measures analysis of variances (rmANOVA) for each EEG measure were carried out. The ERP amplitude, theta ERO, and theta PLF ANOVAs consisted of three within-group factors, including drug (placebo and ketamine), deviant type (frequency, intensity, vowel duration, across vowel, and consonant) and electrode site [left (F3), central (Fz), and right (F4)]. A similar but secondary set of analyses, limited to ERP amplitude, were conducted using responses derived at left (TP9) and right (TP10) temporal electrode sites. MMN latency (at Fz only) was analyzed with similar ANOVAs but with no site factor, while latency at temporal sites was analyzed at left and right hemispheres. Finally, for ERPs, the N100 amplitude/latency values derived from the standard stimulus were also subjected to similar rmANOVA with drug and electrode site (Fz, F3, and F4) as factors. The eLORETA-derived current density values were also analyzed using a rmANOVA and involved two within-group factors, including drug (placebo and ketamine) and ROI (BA41, BA42, BA44, BA45, and BA47). Significant (Greenhouse-Geisser corrected when appropriate) effects were followed up with Bonferroni-adjusted comparisons. The CADSS subscales exhibited non-normal distributions and were analyzed using the non-parametric Wilcoxon Signed Ranks Test (WSRT).

Spearman’s rho correlations were used to examine the relationship between baseline (placebo) electrophysiological measures (ERP, ERO, PLF, and BA current density) with changes in dissociate/perceptual symptoms [difference scores (ketamine – placebo) as for the CADSS sub-scales]. For ERPs (MMN), correlations were only conducted with the frontal midline recording site (Fz) and only correlations equal/less than *P* < 0.01 were considered significant in order to reduce likelihood of chance findings associated with multiple testing.

## Results

### CADSS Symptom Ratings

**FIGURE 2:**
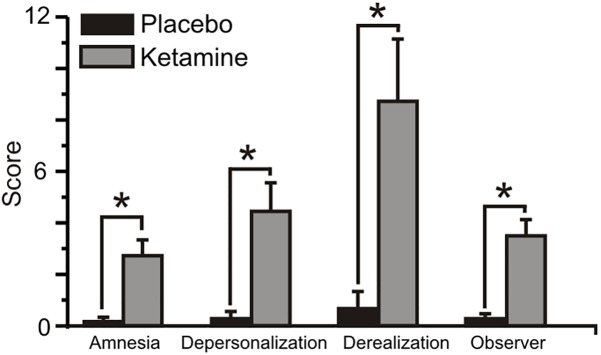
Mean (±SE) Clinician Administered Dissociated States Scale (CADSS) values for the placebo and ketamine conditions. ^∗^*p* < 0.001.

Figure 2 displays the mean (±SE) rating scores for the placebo and ketamine conditions. Relative to placebo, ketamine increased observer rated symptom scores (WSRT = -3.40, *p* < 0.001) and for self-ratings, depersonalization (WSRT = -3.30, *p* < 0.001), derealization (WSRT = -3.52, *p* < 0.0001), and amnesia scores (WSRT = -3.31, *p* < 0.001) were significantly higher with ketamine than with placebo.

### MMN Amplitude/Latency

**FIGURE 3:**
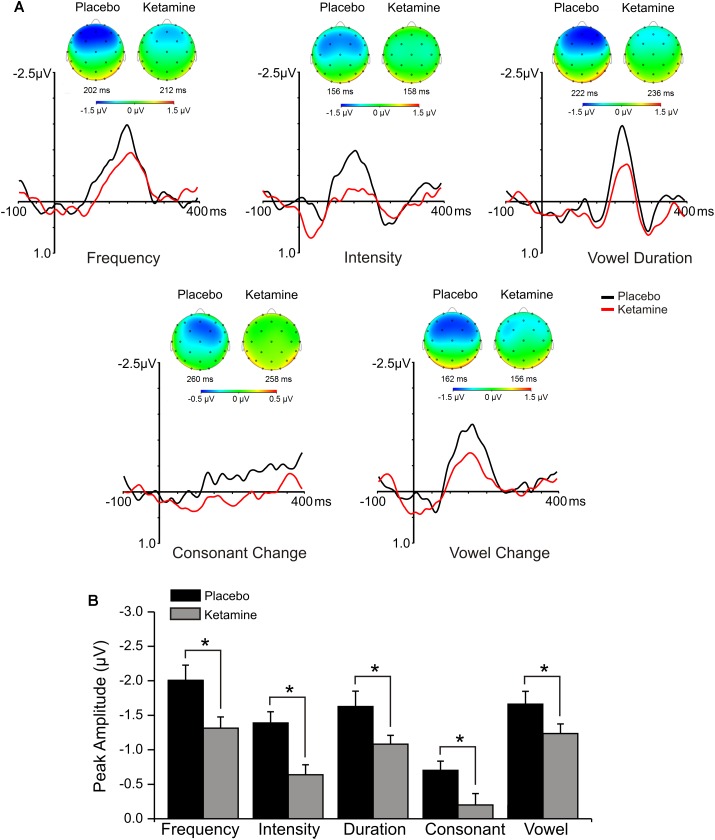
**(A)** Grand averaged ERP waveforms at Fz and topographic maps generated in response to 5 speech deviants during placebo and ketamine conditions. **(B)** Mean (±SE) peak frontal amplitudes (μV) for the placebo and ketamine conditions. ^∗^*p* < 0.05.

Mid-frontal (Fz) grand averaged deviant-minus-standard difference waveforms for each deviant type in the multi-feature speech paradigm presented in placebo and ketamine conditions are shown in Figure 3. Significant main effects were observed for the drug condition across all linguistic sound features (*F* = 24.29, df = 1/17, *p* < 0.0001), with ketamine reducing MMN in response to changes in syllable frequency, syllable intensity, vowel-duration, consonant change, as well as vowel change. Drug did not interact with deviant type or electrode site, but follow-up of a significant deviant effect (*F* = 18.17, df = 4/68, *p* < 0.0001) found largest MMNs with frequency, vowel duration and across vowel deviants, which showed similar amplitudes, each of which was greater than intensity (*p* = 0.01), with the latter deviant producing the smallest of the MMNs. For the significant electrode site effect (*F* = 8.39, df = 2/34, *p* < 0.001), MMN amplitude at frontal midline (Fz) was greater than both left (F3) and right (F4) frontal MMNs (*p* < 0.05), which exhibited similar amplitudes. Frontal MMN latency was not altered by ketamine.

**FIGURE 4:**
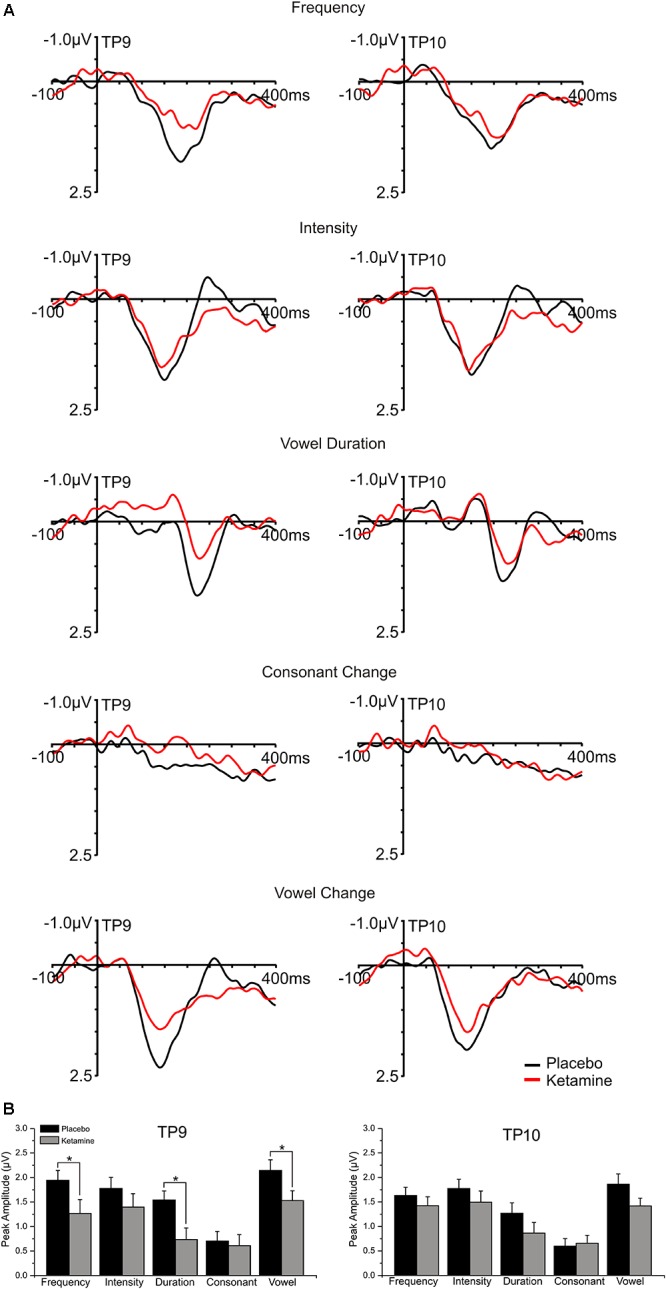
**(A)** Grand averaged ERP waveforms at mastoid sites (TP9 and TP10) generated in response to 5 speech deviants during placebo and ketamine conditions. **(B)** Mean (±SE) peak temporal amplitudes (μV) for the placebo and ketamine conditions. ^∗^*p* < 0.05.

Left (TP9) and right (TP10) temporal grand averaged deviant-minus standard waveforms for each deviant type recorded during placebo and ketamine conditions are shown in Figure 4. Although not interacting with deviant type or electrode site, a significant drug effect (*F* = 6.17, df = 1/17, *p* < 0.03) showed reduced MMN amplitudes with ketamine vs. placebo administration, particularly at TP9 sites. For the significant deviant type effect (*F* = 10.19, df = 4/68, *p* < 0.0001), the largest MMNs were equivalently elicited by intensity, frequency and across vowel deviants. Compared to all deviant types, the consonant deviant elicited the smallest MMN (*p* < 0.001). MMN elicited by the duration deviant was similar to the MMN elicited by the frequency deviant but smaller than the intensity (*p* < 0.04) and vowel duration (*p* < 0.007) MMN. Temporal MMN latencies were not affected by ketamine.

N1 amplitude/latency derived from the standard stimulus ERP frontal (Fz) recording sites were not altered by ketamine administration (*F* = 0.76, df = 1/17, *p* = 0.79).

### Evoked Theta Power

**FIGURE 5:**
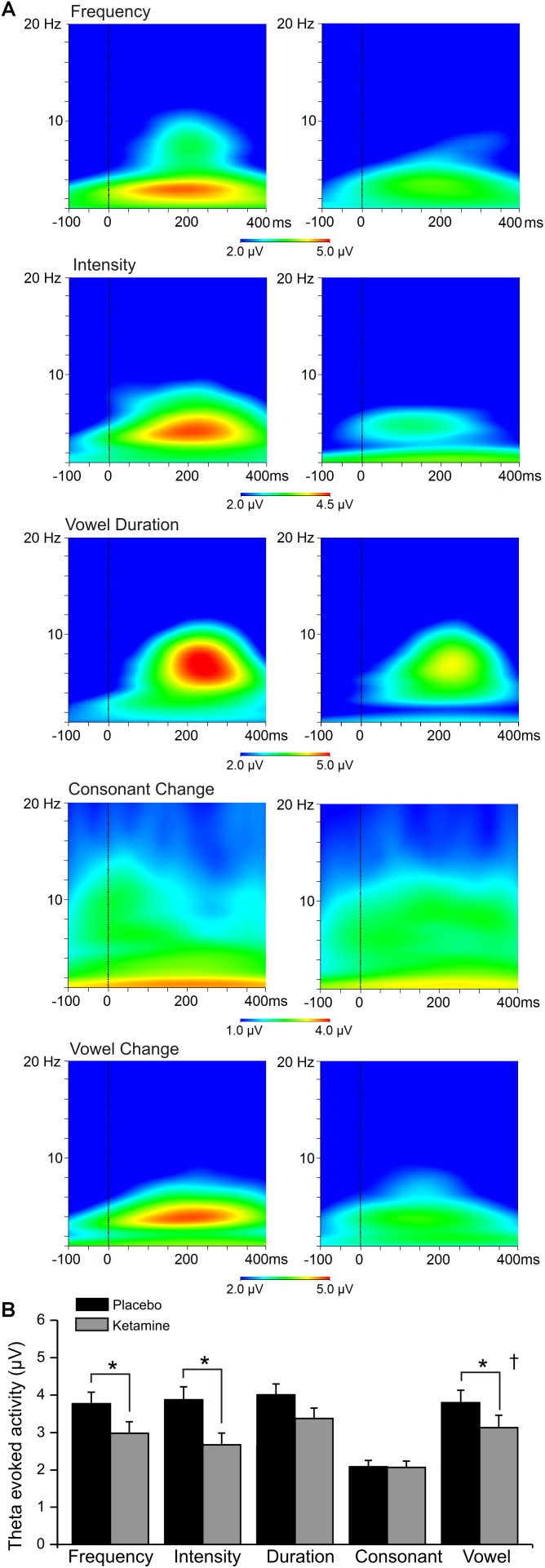
**(A)** Grand averaged time frequency plots showing theta power response at the frontal midline (Fz) site to 5 speech deviants during placebo and ketamine conditions. **(B)** Mean (±SE) frontal theta evoked activity (μV) for the placebo and ketamine conditions. ^∗^*p* < 0.05. † = at Fz only.

Figure 5 shows the grand averaged evoked (stimulus-locked) time-frequency responses to each speech deviant type during placebo and ketamine administration sessions. Significant main drug effects were exhibited (*F* = 21.99, df = 1/17, *p* < 0.0001), but follow-up tests showed evoked theta power for frequency, intensity and vowel deviants being reduced by ketamine compared to placebo administration. Although not interacting with deviant type, follow-up of a significant deviant effect (*F* = 9.35, df = 4/68, *p* < 0.001) found greater evoked power at midline (Fz) compared to both left (F3) and right (F4) sites (*p* < 0.007). Evoked theta power elicited by the standard stimulus was not affected by ketamine. Ketamine administration did not alter ongoing spontaneous theta power derived from frontal recording sites.

### Phase Locking Factor

**FIGURE 6:**
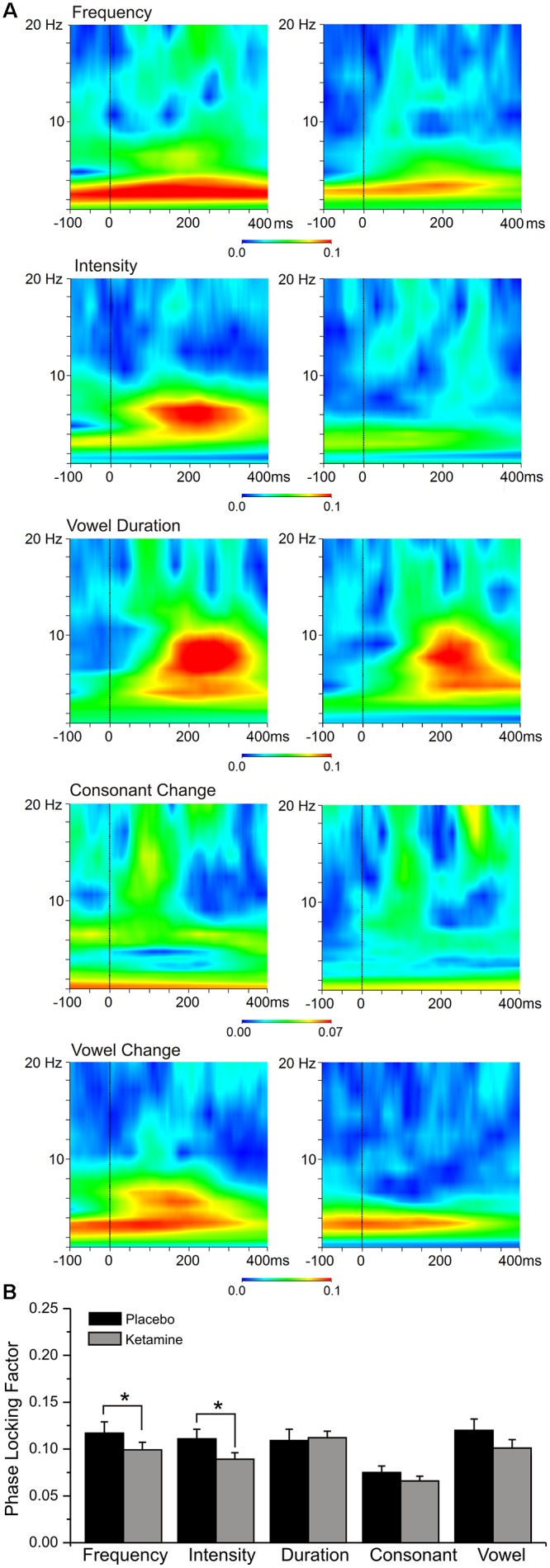
**(A)** Grand averaged event-related spectral perturbation (ERSP) responses highlighting theta phase locking factor changes at the frontal midline (Fz) site to 5 speech deviants in placebo and ketamine conditions. **(B)** Mean (±SE) frontal phase locking factor for the placebo and ketamine conditions.

Grand averaged PLF for each speech deviant type during placebo and ketamine conditions are shown in Figure 6. Significant overall drug effects were observed (*F* = 9.63, df = 4/68, *p* < 0.0001) with ketamine (vs. placebo) significantly diminishing PLF in relation to frequency and intensity deviant types. A significant deviant effect (*F* = 8.13, df = 4/68, *p* < 0.0001) showed consonant PLF to be smaller compared to PLFs associated with other deviants (*p* < 0.02). Electrode site differences were also shown (*F* = 3.49, df = 2/34, *p* < 0.05), with greater PLF being observed at frontal midline (Fz) site compared to left (F3) and right (F4) frontal sites (*p* < 0.007). Theta evoked power elicited by the standard stimuli was not influenced by ketamine, nor was PLF derived from spontaneous (pre-stimulus) activity affected by ketamine.

### Source Current Density

**FIGURE 7:**
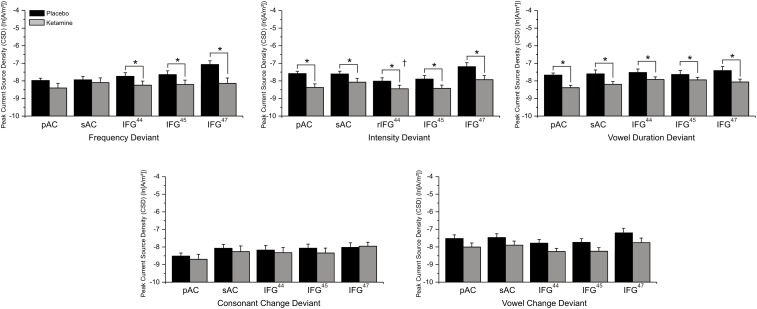
Mean (±SE) ln-transformed source-localized CSD values (A/m^2^) of Brodmann Area (BA) regions of interest associated with 5 speech deviants during placebo and ketamine conditions. ^∗^*p* < 0.05 (IFG^44^ = BA44; IFG = BA45; IFG^47^ = BA47; † = right hemisphere effect).

Mean (±SE) ROI current density values for auditory and frontal cortices during placebo and ketamine conditions are displayed in Figure 7. With the exception of consonant and vowel change deviants, current density across all deviants and ROIs were significantly altered by drug administration (*F* = 13.03, df = 1/17, *p* < 0.002), with ketamine reducing current density vs. placebo. Only the pAC and sAC regions failed to respond to ketamine during the processing of frequency deviants. Regions varied in current density (*F* = 16.94, df = 4/68, *p* < 0.0001), with the highest current density being observed in the IFG (BA47) compared to other regions (*p* < 0.003) and the lowest density was in the pAC.

In a significant deviant effect (*F* = 3.19, df = 4/68, *p* < 0.02), consonant change produced the lowest current density compared to other deviants (*p* < 0.02) and, although similar to vowel duration and syllable intensity/frequency deviant current densities, the across vowel deviant exhibits the highest current density values. Follow-up of a significant deviance x region interaction (*F* = 4.02, df = 16/272, *p* < 0.0001), found that the comparatively lower consonant (vs. other deviants) current density was limited to the pAC (*p* < 0.02) and that for all deviants the highest current density was in the IFG (BA47), which exhibited greater density values than the pAC across deviants (*p* < 0.05), greater density values than all other regions for the vowel duration deviant (*p* < 0.04), and greater density values than BA44 (*p* < 0.04) and BA45 (*p* < 0.03) for syllable frequency, syllable intensity and across vowel deviants.

### Neural-CADSS Correlations

**FIGURE 8:**
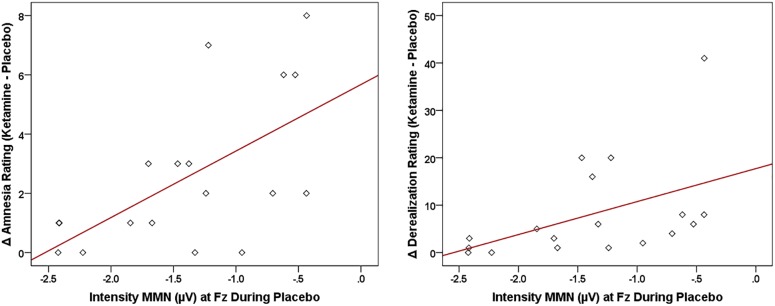
Scatterplots showing significant relationships between ketamine-induced changes in symptom rating (derived by subtracting placebo CADSS values from ketamine CADSS values) and baseline (placebo) intensity MMN amplitude.

**FIGURE 9:**
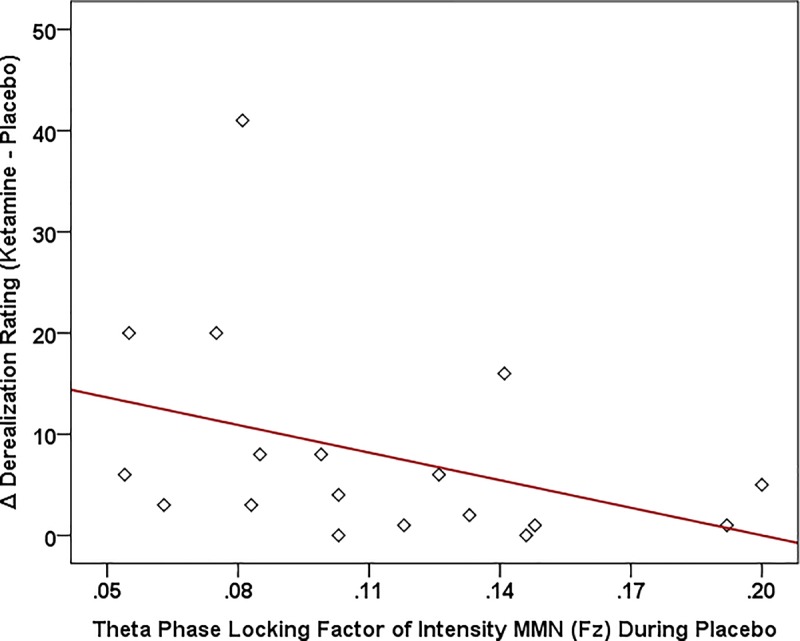
Scatterplot showing significant relationship between ketamine-induced changes in symptom rating (derived by subtracting placebo CADSS values from ketamine CADSS values) and baseline (placebo) and theta phase locking factor (PLF).

Of the three speech MMNs, which were reduced in amplitude with ketamine administration, only the syllable intensity MMN and its associated oscillatory and source activities were related to changes in subjective symptoms elicited by ketamine administration (Figure 8). With respect to MMN itself, individuals with smaller Fz amplitudes during placebo experienced greater ketamine-induced increases in derealization (*r* = 0.631, *N* = 18, *p* < 0.001) and amnesia ratings (*r* = 0.594, *N* = 18, *p* < 0.009). As shown in Figure 9, placebo theta PLF at Fz were negatively correlated with ketamine-induced increases in derealization (*r* = -0.613, *N* = 18, *p* < 0.007) ratings.

**FIGURE 10:**
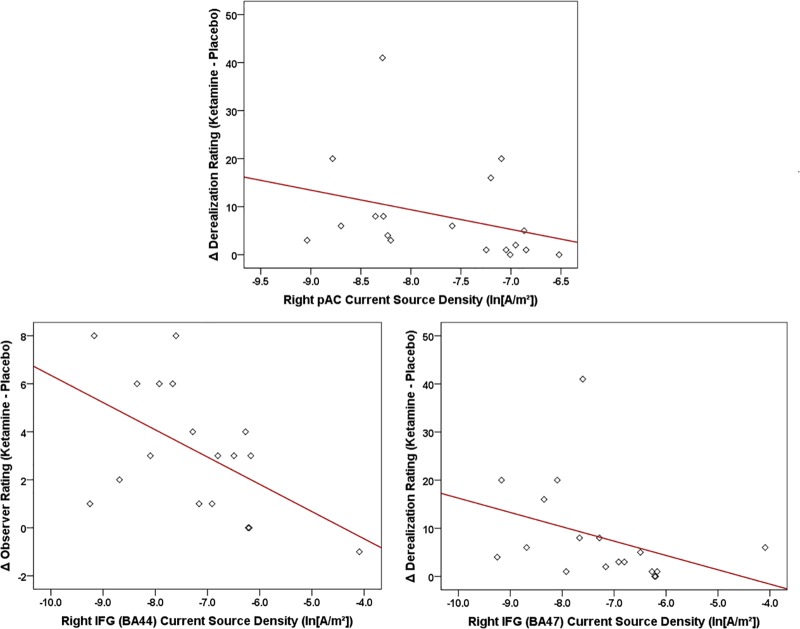
Scatterplots showing significant relationships between ketamine-induced changes in symptom rating (derived by subtracting placebo CADSS values from ketamine CADSS values) and baseline (placebo) current density in LORETA-sources regions of interest.

For the LORETA-derived ROIs (Figure 10), current density in the right hemisphere pAC was inversely related to changes in amnesia (*r* = -0.612, *N* = 18, *p* < 0.007) and derealization ratings (*r* = -0.608, *N* = 18, *p* < 0.007) with ketamine, while current density in regions of interest of the right IFG were negatively correlated with ketamine-induced increases in self-rated derealization (BA47: *r* = -0.584, *N* = 18, *p* < 0.01) and observer-rated psychotomimetic symptoms (BA44: *r* = -0.555, *N* = 18, *p* < 0.017).

## Discussion

### Summary of Findings

The present study evaluated the hypothesis that the NMDA receptor system is critically involved in the early sensory processing of speech. As predicted, a sub-anesthetic dose of the NMDA antagonist, ketamine, significantly diminished frontal and temporal MMN generation in response to speech deviants. To our knowledge, this is the first report suggesting a causative relationship between NMDA receptor blockade and impaired speech MMN generation. The absence of ketamine effects on sensory ERP (N1) with a similar latency suggests that the reduction of speech MMNs in this study is not the result of a general weakening of ERP generators, but specifically involves neuronal processes generating the auditory MMN, including the MMN elicited by human speech. Supporting this, NMDA receptor antagonism reduced both evoked theta power and theta phase locking associated with diminished speech MMNs, and it attenuated current density in the auditory (pAC and sAC) cortex and in IFG brain regions involved in the processing and production of speech. This is consistent with previous studies showing a contributing influence of theta oscillations, and activity in temporal and frontal brain regions in the generation of the MMN. In our healthy volunteers without a history of psychopathology, speech associated neural activity assessed in the non-drug (placebo) state was negatively correlated with ketamine-induced psychotomimetic experiences, indicating that these electrophysiological markers may reflect the functional state of the NMDA receptor-mediated system and its vulnerability to disruption by receptor antagonism.

### Ketamine-MMN Effects

Though no ketamine influence was observed on MMN latency, our MMN amplitude results with speech MMNs in healthy volunteers generally conform with meta-analyses involving simple tone stimuli in humans, which show reductions in the MMN response with ketamine (Rosburg and Kreitschmann-Andermahr, 2016). This effect is also reported in a range of animal models, including recordings in mice (Umbricht et al., 2005; Ehrlichman et al., 2008), rats (Tikhonravov et al., 2008), monkeys (Javitt et al., 1996; Gil-da-Costa et al., 2013), and also pigeons (Schall et al., 2015). Whereas in previous research ketamine’s effects on MMN generated by simple acoustic stimuli were numerically but not statistically different for deviant type (i.e., ketamine tended to attenuate tone duration MMN amplitude more than frequency MMN amplitude on visual inspection alone, but this did not reach statistical significance), ketamine impairment of speech MMNs was observed across all deviant types at frontal and less so at temporal recordings. Although MMN amplitudes varied with speech deviance, the baseline (placebo) temporal MMN amplitude generally did not appear to moderate the presence of significant ketamine effects on MMN. For example, MMN in response to consonant change was significantly smaller than MMNs elicited by the other four but all were significantly affected by ketamine administration. The noticeably small consonant MMN, exhibiting no clear discernible onset/offset or peak within the typical MMN latency window, has been attributed, in previous work, to more difficult discriminability (vs. standard) based on behavioral discrimination tests (Kantrowitz et al., 2015).

Mismatch negativity is thought to consist of two main generators: one in the bilateral auditory cortex, underlying pre-perceptual sound change detection in the auditory cortex, which is thought to trigger the frontal cortex MMN generator, associated with the initiation of attention switch to sound change (Näätänen and Alho, 1997; Näätänen et al., 2007). Given the ketamine-induced MMN reductions at frontal and temporal recording sites and the suggestion that MMN generation involves prediction-error signaling (Todd et al., 2013), it is reasonable to speculate that in the language domain, glutamate dysregulation due to NMDA receptor hypofunction may induce problems in prediction-error generation (i.e., the MMN) in a temporo-frontal network circuit involved in early sensory processing, manifesting as diminished MMNs to speech deviants. Non-significant ketamine effects for consonant and syllable intensity deviant MMNs at temporal but not frontal cortex has potential relevance for MMN findings in schizophrenia.

### Ketamine-Oscillatory Effects

In addition to our MMN observations using the conventional time-domain approach, we also adopted the approach of more recent human studies and utilized ‘time-frequency’ neuro-oscillatory analyses of electrophysiological responses as a function of underlying spectral frequency.

To our knowledge, this is the first investigation of the time-frequency signature of human MMN responses to NMDA receptor antagonist effects. Not observed with the standard stimulus, averaged evoked theta power and theta phase locking associated with speech (frequency, intensity, vowel) deviants were found to be significantly diminished by ketamine compared to placebo. Time-frequency studies have revealed the contribution of the theta frequency band in driving the neuronal generation of the MMN in frontal and temporal areas. Although this research shows that neural generation of the MMN response is accompanied by theta band power modulation and phase alignment, their individual contribution varies with deviant type (Lee et al., 2017) and their specific association with normal and abnormal sensory/cognitive processes is not clear. NMDA receptor antagonists have been found to reduce evoked theta power during MMN generation (Lee et al., 2018) and other task events in rodent models (Ehrlichman et al., 2009; Lazarewicz et al., 2009). Interestingly, speech-evoked MMN and theta power in healthy volunteers serve as predictors of behavioral speech perception at the syllable and sentence level and, along with perception accuracy are reduced during noise stress (Koerrner et al., 2016), which also disrupts NMDA receptor signaling (Cui et al., 2012).

Phase resetting, which refers to the systematic adjustment of the phase of ongoing oscillations to give a consistent phase relationship to a stimulus, contributes to the generation of ERPs including the MMN. Indexed by the PLF measure of inter-trial phase variability, modulation of theta phase consistency across trials of oscillatory neural activity was significantly reduced with ketamine administration. These effects, observed in relation to frequency and intensity deviants, were concurrent with ketamine-induced reduction of speech MMNs and indicate that NMDA receptor-mediated neurotransmissions and NMDA receptor hypo-function underly normal and abnormal theta phase resetting dynamics contributing to speech MMN generation in healthy volunteers, Rodent MMN and evoked theta power impaired by NMDA receptor antagonism are mitigated by concurrent treatment with the NMDA receptor agonist glycine, suggesting that MMN generation both in time (ERP) and time-frequency (neuro-oscillatory) domain analyses, may serve as effective translational measures for revealing circuit dynamics underlying acoustic deviance detection. Compared to the auditory high frequency gamma (40 Hz) response, deficits of which are associated with impaired function of rapidly firing cortical parvalbumin (PV)-type expressing inhibitory interneurons in thalamocortical circuits (Gonzalez-Burgos et al., 2011), theta frequency rhythms are linked to interactions involving more slowly firing non-PV cells, especially somatostatin-type (SST) inhibitory interneurons in cortical circuits (Womelsdorf et al., 2014).

It is of relevance to note that ketamine-induced changes in evoked theta and PLF occurred in the absence of any changes in background, ongoing theta oscillations. Analysis of baseline (pre-stimulus) and resting EEG in healthy humans administered ketamine has been mixed and have not consistently mimicked this oscillatory abnormality (Knott et al., 2006; Kocsis et al., 2013; de la Salle et al., 2016). These present findings, observed with a sub-anesthetic dose of ketamine, suggest that impaired detection of speech deviants during NMDA receptor blockade may not be dependent on alterations in tonic cortical activity.

### Ketamine-Source Effects

Neural activity in ROIs involved in the temporofrontal network implicated in the generation of the auditory MMN elicited by speech (frequency, intensity, vowel duration) change was significantly attenuated by ketamine. Depending on deviant type, intracortical current density in both temporal and frontal cortical areas was reduced concurrently with impairments in scalp-recorded MMN and theta oscillatory responses during detection of speech deviants. Paralleling recent LORETA observations in healthy volunteers of ketamine-induced reductions in current density of auditory, cingulate and middle frontal cortical sources of tone deviant MMNs (Thiebes et al., 2017), our present source localization findings support a role for deficient NMDA receptor-mediated neurotransmission in hypofunction of auditory and frontal cortical generators associated with aberrant speech deviance detection in schizophrenia.

Within the predictive coding formulation of MMN generation, which accommodates the findings of multiple studies showing that there are temporal and frontal cortical sources underlying MMN generation (Garrido et al., 2009b), our findings of ketamine-induced reductions in speech MMNs together with current density reductions in temporal and frontal cortical regions may indicate alterations in prediction error in low level (auditory) sources and in higher cortical levels. Additionally, they may reflect changes in temporal-frontal connections that are thought to involve NMDA receptor-dependent synaptic plasticity and underly MMN generation (Garrido et al., 2008, 2009a). Data from neuroimaging studies of auditory-change detection suggest that recruitment of the frontal cortex (IFG), particularly in the right hemisphere, increases with decreasing deviance. This suggests that the right IFG may engage with the auditory cortex to produce a contrast enhancing effect facilitating the detection of smaller deviances (Opitz et al., 2002; Doeller et al., 2003), or complex (e.g., language), ambiguous (e.g., Finnish stimuli), and difficult-to-detect stimulus changes. Although the typically observed sequential activation of these sources (auditory cortical preceding frontal) is compatible with the notion that the auditory cortical output is “evaluated” in some way by frontal regions (Todd et al., 2012) and is observed in response to large deviants, a reverse activation pattern (IFG activation before temporal) is observed with the processing of small deviants (Tse et al., 2013). Interestingly, strong evoked theta band synchronization between temporal and frontal regions is observed during auditory MMN generation (Hsiao et al., 2010; Choi et al., 2013).

### Relevance to Schizophrenia

Ketamine-induced reductions in speech MMNs at frontal and temporal recordings mimic the impaired speech MMN generation observed in schizophrenia with EEG and magnetoencephalography (MEG) recordings (Kasai et al., 2002; Kasai, 2004; Yamasue et al., 2004; Kawakubo et al., 2006; Kawakubo et al., 2007; Fisher et al., 2008) and directly implicate deficient NMDA receptor- dependent neurotransmission in the aberrant processing of speech in this disorder. NMDA-mediated disruption of speech processing may possibly contribute to negative symptoms (such as social withdrawal) by reducing attentional switching to socially relevant auditory cues (e.g., intensity in a speaker’s voice) and/or may contribute to the phenomenology of thought-disordered speech and false perceptual influences in the language system (i.e., auditory verbal hallucinations).

These MMN effects were concurrent with ketamine-induced disruptions in evoked theta power and theta phase locking and are consistent with findings linking MMN dysfunction in chronic and first episode schizophrenia primarily to an impaired theta frequency response (Todd et al., 2008; Bishop and Hardiman, 2010; Moran and Hong, 2011; Choi et al., 2013; Hermann et al., 2014; Xiong et al., 2019). It has been suggested that by diminished theta power modulation and phase resetting in response to speech stimuli, may contribute to cognitive dysfunction in schizophrenia, and may be linked to the impaired interplay between cortical pyramidal neurons and circuit SST-type GABAergic interneurons (Javitt et al., 2018).

These sensor-level neuroelectric alterations with ketamine were paralleled by reductions in intra-cortical current density in frontal and temporal ROIs, cortical areas which have also been shown by either EEG (Hirayasu et al., 1998; Kreitschmann-Andermahr et al., 1999; Pekkonen et al., 2002; Ahveninen et al., 2006; Thonnesen et al., 2008), high-density EEG (Park et al., 2002; Youn et al., 2003), fMRI (Wible et al., 2001; Kircher et al., 2004), or LORETA sourcing (Miyanishi et al., 2013; Takahashi et al., 2013; Fulham et al., 2014), to be underactivated during MMN generation. Weaker frontal/temporal circuitry has also been associated with MMN reductions to speech stimuli in schizophrenia (Thiebes et al., 2017), and based on our findings, may implicate deficient NMDA receptor-mediated neurotransmission in underlying frontal and auditory cortical generators.

The clinical findings with ketamine in healthy volunteers were as expected, with significant dissociative effects reflected in marked increases in self-rated derealization scores, as well as by increases in amnesia and observer-rated scores. Consistently reported, and co-occurring with reductions in MMN in previous ketamine studies, these perceptual/dissociative phenomena are part of a spectrum of transient schizophrenia-like psychoactive effects (including positive and negative symptoms) produced with acute sub-anesthetic ketamine (Thiebes et al., 2017). Unrelated to baseline symptomatology, ketamine also exacerbates positive symptoms in schizophrenia patients in a way that is strikingly reminiscent of symptoms during active episodes of the disorder (Lahti et al., 2001). Although not blocked by conventional antipsychotics such as haloperidol, and only blunted by the atypical antipsychotic clozapine (Malhotra et al., 1997), clinical trials with sarcosine (a glycine reuptake inhibitor) and glycine site agonists (e.g., D-serine) have reported beneficial effects on the negative symptoms of schizophrenia (Kircher et al., 2004; Miyanishi et al., 2013) (both glycine and glutamate binding are essential for NMDA receptor activation). Active high-dose glycine attenuates MMN in healthy humans (Leung et al., 2008) but clinical trials with D-serine in schizophrenia showed acute increases in duration MMN. Further, chronic (6 weeks) increases in frequency MMN were observed, as well as improvements in positive and negative symptoms, both of which were predicted by baseline MMN amplitudes (i.e., smaller amplitudes correlated with greater clinical response) (Kantrowitz et al., 2016, 2018; Greenwood et al., 2018). Relatedly, glutathione deficiency also produces schizophrenia phenotypes including hypofunction of NMDA receptors (Steullet et al., 2006), and administration of the glutathione precursor *N*-acetyl-cysteine (NAC) improved MMN in chronic schizophrenia (Lavoie et al., 2008) and in early psychosis (Retsa et al., 2018).

Although not implying any risk of disorder onset in our healthy volunteers, individuals varied in their susceptibility to particular psychotomimetic symptoms induced by ketamine. Individual differences in ketamine-induced effects were strongly correlated with the magnitude of MMN in a baseline (placebo) condition, in particular with smaller amplitudes to the intensity deviant being associated with more intense psychosis-like symptoms. This finding parallels similar observations showing baseline tone duration and tone frequency MMNs correlating with the degree of psychosis-like experiences with ketamine (Umbricht et al., 2002). Also shown with the intensity deviant following ketamine infusion, evoked theta power and theta phase locking, as well as intracortical current density in auditory (pAC) and language-related (BA44 and BA47) brain regions at baseline were inversely correlated with the degree of psychotomimetic experiences. As with previous fMRI studies, in which activity of specific symptom changes correlated with functional alterations in distinct brain regions during ketamine infusion (Deakin et al., 2008; Honey et al., 2008), selective theta oscillatory indices and regional current densities exhibited differing relationships to ketamine-induced psychotomimetic experiences, thus supporting a NMDA receptor-regulated neural basis for the varied symptoms characterizing schizophrenia. Baseline individual differences in MMN-indexed vulnerability to psychosis may reflect genetic influences on the cortical expression of glutamate receptors, which are reduced in schizophrenia (Catts et al., 2016) and have been shown to modulate tonal (Featherstone et al., 2015) and phonetic (Kawakubo et al., 2011) MMN response.

As observed in earlier work (Stone et al., 2008), the degree of psychosis-like experiences evoked by NMDA receptor antagonists varies with the degree of NMDA receptor occupancy. As such, stronger psychotomimetic responses at the same dose of NMDA receptor antagonist reflect a greater disruption in NMDA receptor-dependant neurotransmission and, accordingly, the MMN has been interpreted as providing some information about the functional “condition” of NMDA receptor-dependent systems. This can be regarded as an indicator of the vulnerability or resiliency of the NMDA receptor system to acute perturbation by antagonists: a smaller MMN indicates a less resilient or less abundant NMDA receptor system, that is also more perturbed by NMDA receptor antagonist treatment (Umbricht et al., 2002). Similarly, speech-related MMNs and their associated theta oscillations and intracortical current densities in frontotemporal regions may serve as neural indicators of the functional state of NMDA receptor-mediated neurotransmission and may be useful neuroelectric tools in studies of normal and abnormal NMDA receptor functioning in humans.

The reasons why these relationships between baseline (placebo) electrophysiology and symptom changes with ketamine were limited to detection of syllable intensity deviants are not clear. Impaired sensitivity to changes in pitch and duration (Todd et al., 2003; Gold et al., 2012) processes known to localize to the primary auditory cortex (Tramo et al., 2002), along with deficits in sensitivity to modulation of intensity discrimination are frequently observed in schizophrenia (Bach et al., 2011). In studies of auditory emotion recognition, patients have also shown relatively greater deficit in the ability to use pitch-based versus absolute intensity-based features of speech (Leitman et al., 2010a; Gold et al., 2012). Although it is difficult to compare the degree of change across speech deviants in our study, acoustic/vocal stimuli with high cue saliency tend to increase temporal cortex activation and associated sensory-integrative functions, while with ambiguous, low salience speech stimuli, greater evaluative processes associated with inferior temporal regions are recruited (Leitman et al., 2010b). It is possible that our baseline MMN-ketamine response associations seen with intensity deviants may reflect the unique cortical recruitment pattern specific to changes in speech intensity and its disruption by NMDA receptor hypofunction.

## Limitations

These results provide potential insight into some of the neural mechanisms underlying NMDA receptor-mediated impairments in the sensory processing of human speech, but there are limitations to the study. The participant sample was limited to young, adult (English-speaking) healthy male volunteers, who tend to exhibit greater acute vulnerability (vs. females) to some of the behavioral effects of ketamine (Morgan et al., 2006), and thus the findings do not necessarily translate to schizophrenia patients who develop NMDA receptor adaptation with increasing chronicity. Also, although their age range (early twenties) is comparable to the typical age of onset of schizophrenia, findings may not translate for speech processing in chronic patients. New language-specific neural representations (indexed by MMN) can evolve for non-native speech categories (Tamminen et al., 2015), and although the MMN was elicited within an optimal paradigm which allowed for neural profiling across different speech features, they were recorded in response to deviations in Finnish-language speech sounds and similar studies relevant to the native language (English) of the test subjects are required. Subjective effects of ketamine were evaluated with an instrument emphasizing perceptual/dissociative symptoms and did not address the positive, negative or cognitive symptomatology that characterizes schizophrenia. Ketamine was administered as a single dose, with no attempt to examine dose- or time-response variations, and the design lacked an active comparator drug (e.g., midazolam) which may allow for the assessment of non-specific behavior (e.g., drowsiness), and effects unrelated to NMDA receptor blockade. Examination of EEG oscillations was limited to theta frequencies and future studies need to assess a more complete frequency spectrum from slow delta to fast gamma rhythms. Although gamma band oscillations have also been associated with depression and fast-acting antidepressants like ketamine (Fitzgerald and Watson, 2018), they have been frequently associated with the pathophysiology of schizophrenia and associated auditory evoked response deficits (Curic et al., 2019). Moreover, relative to standard sounds, deviant sounds elicit a cascade of oscillatory modulations (beginning with gamma and followed by beta, both of which are coupled with theta) that are altered by acute ketamine administration (Gilley et al., 2017; Schuelert et al., 2018). Further, EEG source localization lacks the spatial sensitivity of other imaging methodologies such as fMRI and was limited to specific regions of interest in the temporal and inferior frontal cortex, thus, it did not include examination of a possible role for other circuits (e.g., thalamocortical and limbic) involved in sensory processing. Although the NMDA receptor is known to specifically influence activity in the central auditory system (Wang et al., 1987) and frontal/limbic and cingulate regions (Vollenweider et al., 1987; Lahti et al., 1995; Northoff et al., 2005), it is also thought to participate in widespread suppression and activation of circuit-level neural activity (Fitzgerald, 2012). Combining EEG and other imaging tools in a ketamine model and assessing activity and connectivity within and between multiple neural networks during stimulus-evoked and resting-state conditions may be a useful approach in future studies for delineating extrinsic and intrinsic neural factors mediating region-specific underpinnings of NMDA receptor-dependent speech/language deficits pertaining to schizophrenia and associated auditory hallucinatory experiences (Northoff and Qin, 2011; Northoff, 2014; Northoff, 2015; Alderson-Day et al., 2016).

## Conclusion

To the best of our knowledge, this is the first study assessing the effects of the NMDA receptor antagonist ketamine on speech MMNs, and their associated oscillatory activities and intra-cortical current densities in healthy humans. Reductions in MMN generation and related neural activity across speech deviants indicates a general impairment in the processing of deviant speech. Together with the emergence of perceptual/dissociative symptoms, these findings generally support a glutamatergic/NMDA receptor model of schizophrenia and of language impairment in schizophrenia in particular. In this non-psychopathological sample, baseline (placebo) MMN, theta oscillations and current density in frontotemporal regions were associated with symptom response to ketamine and may serve as objective indicators of the functional state of NMDA receptor-mediated neurotransmission.

## Ethics Statement

This study was fully reviewed and approved by the Research Ethics Board (REB) of the Royal Ottawa Mental Health Centre. Participants were made aware of potential adverse events, were encouraged to ask questions, and reviewed and signed an informed consent form prior to participating in the study. Participants were also ensured that they could stop study participation at any time for any reason without penalty.

## Author Contributions

VK: conception and design of the project. SdlS, JC, DS, HB, and VI: participant screening. SdlS, JC, DS, HB, and JM: performance of the experiments. SdlS: electrophysiology and statistical analysis. VK and SdlS: interpretation and manuscript preparation.

## Conflict of Interest Statement

The authors declare that the research was conducted in the absence of any commercial or financial relationships that could be construed as a potential conflict of interest.

## References

[B1] Ahveninen J. Jaaskelainen I. Osipova D. Huttunen M. Ilmoniemi R. Kaprio J. (2006). Inherited auditory-cortical dysfunction in twin pairs discordant for schizophrenia. *Biol. Psychiatry* 60 612–620. 10.1016/j.biopsych.2006.04.015 16876141

[B2] Alderson-Day B. Diederen K. Fernyhough C. Ford J. Horga G. Marqulies D. (2016). Auditory hallucinations and the brain’s resting-state networks: findings and methodological observations. *Schizophr. Bull.* 42 1110–1123. 10.1093/schbul/sbw078 27280452 PMC4988751

[B3] Avissar M. Javitt D. (2018). Mismatch negativity: a simple and useful biomarker of N-methyl D-aspartate receptor (NMDAR)-type glutamate dysfunction in schizophrenia. *Schizophr. Res.* 191 1–4. 10.1016/j.schres.2017.11.006 29132813

[B4] Avissar M. Xie S. Vail B. Lopez-Calderon J. Wang Y. Javitt D. (2018). Meta-analysis of mismatch negativity to simple versus complex deviants in schizophrenia. *Schizophr. Res.* 195 25–34. 10.1016/j.schres.2017.07.009 28709770 PMC5745291

[B5] Bach D. Buxtorf K. Strik W. Neuhoff J. Seifritz I. (2011). Evidence for impaired social intensity processing in schizophrenia. *Schizophr. Bull.* 37 426–431. 10.1093/schbul/sbp092 19729389 PMC3044622

[B6] Bickel S. Javitt D. (2009). Neurophysiological and neurochemical animal models of schizophrenia: focus on glutamate. *Behav. Brain Res.* 204 352–362. 10.1016/j.bbr.2009.05.005 19433116 PMC4561625

[B7] Bishop D. Hardiman M. (2010). Measurement of mismatch negativity in individuals: a study using single-trial analysis. *Psychophysiology* 47 697–705. 10.1111/j.1469-8986.2009.00970.x 20210877 PMC2904495

[B8] Bremner J. Krystal J. Putnam F. Southwick S. Marmar C. Charney D. (1998). Measurement of dissociative states with the clinician-administered dissociative states scale (CADSS). *J. Traum. Stress* 11 125–136. 10.1023/A:1024465317902 9479681

[B9] British Broadcasting Corporation Discovery Channel (Firm) and Warner Home Video (Firm). (2002). *The Blue Planet: Seas of Life.* London: BBC Video.

[B10] Brown M. Kuperburg G. (2015). A hierarchical generative framework of language processing: leaking language perception, interpretation and production abnormalities in schizophrenia. *Front. Hum. Neurosci.* 9:643. 10.3389/fnhum.2015.00643 26640435 PMC4661240

[B11] Carlsson A. Hansson L. Waters N. Carlsson M. (1999). A glutamatergic deficiency model of schizophrenia. *Br. J. Psychiatry Suppl.* 37 2–6. 10.1192/s0007125000293574 10211132

[B12] Catts V. Lai Y. Weickert C. Weickert T. Catts S. (2016). A quantitative review of the postmortem evidence for decreased cortical N-methyl-D-aspartate receptor expression in schizophrenia: how can we link molecular abnormalities to mismatch negativity deficits? *Biol. Psychol.* 116 57–67. 10.1016/j.biopsycho.2015.10.013 26549579

[B13] Choi J. Lee J. Ko P. Lee G. Jung K. Kim K. (2013). Fronto-temporal interactions in the theta-band during auditory deviant processing. *Neurosci. Lett.* 548 120–125. 10.1016/j.neulet.2013.05.079 23769731

[B14] Corcoran C. Stoops A. Lee M. Martinez A. Sehatpour P. Dias E. (2018). Developmental trajectory of mismatch negativity and visual event-related potentials in healthy controls: implications for neurodevelopmental vs. neurodegenerative models of schizophrenia. *Schizophr. Res.* 191 101–108. 10.1016/j.schres.2017.09.047 29033283 PMC5866919

[B15] Coyle J. Balu D. Benneyworth M. Basu A. Roseman A. (2010). Beyond the dopamine receptor: novel therapeutic targets for treating schizophrenia. *Dialogues Clin. Neurosci.* 12 359–382. 20954431 10.31887/DCNS.2010.12.3/jcoyle PMC3181979

[B16] Cui B. Wu M. She X. Liu H. (2012). Impulse noise exposure in rats causes cognitive deficits and changes in hippocampal neurotransmitter signaling and tau phosphorylation. *Brain Res.* 1427 35–43. 10.1016/j.brainres.2011.08.035 22055774

[B17] Curic S. Leicht G. Thiebes S. Andreou C. Polomac N. Eichler I. (2019). Reduced auditory evoked gamma-band response and schizophrenia-like clinical symptoms under subanesthetic ketamine. *Neuropsychopharmacology* . 30758327 10.1038/s41386-019-0328-5 PMC6785009

[B18] de la Salle S. Choueiry J. Shah D. Bowers H. McIntosh J. Ilivitsky V. (2016). Effects of ketamine on resting-state EEG activity and their relationship to perceptual-dissociative symptoms in healthy humans. *Front. Pharmacol.* 7:348. 10.3389/fphar.2016.00348 27729865 PMC5037139

[B19] Deakin J. Lees J. McKie S. Hallak J. Williams S. Dursun S. (2008). Glutamate and the neural basis of the subjective effects of ketamine. *Arch. Gen. Psychiatry* 65 154–164. 10.1001/archgenpsychiatry.2007.37 18250253

[B20] Doeller C. Opitz B. Mecklinger A. Krick C. Reith W. Schroger E. (2003). Prefrontal cortex involvement in preattentive auditory deviance detection: neuroimaging and electrophysiological evidence. *Neuroimage* 20 1270–1282. 10.1016/S1053-8119(03)00389-6 14568496

[B21] Ehrlichman R. Gandal M. Maxwell C. Lazarewicz M. Finkel L. Siegel S. (2009). N-methyl-D-aspartic acid receptor antagonist-induced frequency oscillations in mice recreate pattern of electrophysiological deficits in schizophrenia. *Neuroscience* 158 705–712. 10.1016/j.neuroscience.2008.10.031 19015010

[B22] Ehrlichman R. Maxwell C. Majumdar S. Siegel S. (2008). Deviance-elicited changes in event-related potentials are attenuated by ketamine in mice. *J. Cogn. Neurosci.* 20 1403–1404. 10.1162/jocn.2008.20097 18303985

[B23] Featherstone R. Shin R. Kogan J. Liang Y. Matsumoto M. Siegel S. (2015). Mice with subtle reduction of NMDA NR1 receptor subunit expression have a selective decrease in mismatch negativity: implications for schizophrenia prodromal population. *Neurobiol. Dis.* 73 289–295. 10.1016/j.nbd.2014.10.010 25461194

[B24] Fisher D. Labelle A. Knott V. (2008). Auditory hallucinations and the mismatch negativity: processing speech and non-speech sounds in schizophrenia. *Int. J. Psychophysiol.* 70 3–15. 10.1016/j.ijpsycho.2008.04.001 18511139

[B25] Fitzgerald P. (2012). The NMDA receptor may participate in widespread suppression of circuit level neural activity, in addition to a similarily prominent role in circuit level activation. *Behav. Brain Res.* 230 291–298. 10.1016/j.bbr.2012.01.057 22342923

[B26] Fitzgerald P. Watson B. (2018). Gamma oscillations as a biomarker for major depression: an emerging topic. *Transl. Psychiatry* 8:177. 10.1038/s41398-018-0239-y 30181587 PMC6123432

[B27] Fuentemilla L. Marco-Pollares J. Munte T. Grau C. (2008). Theta EEG oscillatory activity and auditory change detection. *Brain Res.* 1220 93–101. 10.1016/j.brainres.2007.07.079 18076870

[B28] Fulham W. Michie P. Ward P. Rasser P. Todd J. Johnson P. (2014). Mismatch negativity in recent-onset and chronic schizophrenia: a current source density analysis. *PLoS One* 9:e100221. 10.1371/journal.pone.0100221 24949859 PMC4064992

[B29] Garrido M. Friston K. Kiebel S. Stephan K. Baldeweg T. Kilner J. (2008). The functional anatomy of the MMN: a DCM study of the coving paradigm. *Neuroimage* 42 936–944. 10.1016/j.neuroimage.2008.05.018 18602841 PMC2640481

[B30] Garrido M. Kilner J. Kiebel S. Friston K. (2009a). Dynamic causal modeling of the response to frequency deviants. *J. Neurophysiol.* 101 2620–2631. 10.1152/jn.90291.2008 19261714 PMC2681422

[B31] Garrido M. Kilner J. Stephan K. Friston S. (2009b). The mismatch negativity: a review of underlying mechanisms. *Clin. Neurophysiol.* 120 453–463. 10.1016/j.clinph.2008.11.029 19181570 PMC2671031

[B32] Gil-da-Costa R. Stoner G. Fung R. Albright T. (2013). Nonhuman primate model of schizophrenia using a noninvasive EEG method. *Proc. Natl. Acad. Sci. U.S.A.* 120 15425–15430. 10.1073/pnas.1312264110 23959894 PMC3780912

[B33] Gilley P. Uhler K. Watson K. Yoshinaga-Itano C. (2017). Spectral-temporal EEG dynamics of speech discrimination processing in infants during sleep. *BMC Neurosci.* 18:34. 10.1186/s12868-017-0353-4 28330464 PMC5439120

[B34] Gold R. Butler P. Revheim N. Leitman D. Hansen J. Gur R. (2012). Auditory emotion recognition impairments in schizophrenia: relationship to acoustic features and cognition. *J. Psychiatry* 169 424–432. 10.1176/appi.ajp.2011.11081230 22362394 PMC3882084

[B35] Gonzalez-Burgos G. Fish K. Lewis D. (2011). GABA neuron alternations, cortical circuit dysfunction and cognitive deficits in schizophrenia. *Neural. Plast.* 2011:723184. 10.1155/2011/723184 21904685 PMC3167184

[B36] Gratton G. Coles M. Donchin E. (1983). A new method for off-line removal of ocular artifact. *Electroencephalogr. Clin. Neurophysiol.* 55 468–484. 10.1016/0013-4694(83)90135-9 6187540

[B37] Green M. (2006). Impact of cognitive and social cognitive impairment on functional outcomes in patients with schizophrenia. *J. Clin. Psychiatry* 77(Suppl. 2) 2–8. 10.4088/JCP.14074su1c.02 26919052

[B38] Greenwood L. Leung S. Michie P. Green A. Nathan P. Fitzgerald P. (2018). The effects of glycine on auditory mismatch negativity in schizophrenia. *Schizophr. Res.* 191 61–69. 10.1016/j.schres.2017.05.031 28602646

[B39] Hall M. Taylor G. Salisbury D. Levy D. (2011). Sensory gating event-related potentials and oscillations in schizophrenia patients and their unaffected relatives. *Schizophr. Bull.* 37 1187–1199. 10.1093/schbul/sbq027 20363872 PMC3196947

[B40] Harms L. (2016). Mismatch responses and deviance detection in N-methyl-D-aspartate (n.d.) receptor hypofunction and developmental models of schizophrenia. *Biol. Psychol.* 116 75–81. 10.1016/j.biopsycho.2015.06.015 26159809

[B41] Hermann C. Rach S. Vosskuhl J. Strüber D. (2014). Time-frequency analysis of event-related potentials: a brief tutorial. *Brain Topogr.* 27 438–450. 10.1007/s10548-013-0327-5 24194116

[B42] Hirayasu Y. Potts G. O’Donnell B. Kwon J. Arakaki H. Akdag S. (1998). Auditory mismatch negativity in schizophrenia: topographic evaluation with a high-density recording montage. *Am. J. Psychiatry* 155 1281–1284. 10.1176/ajp.155.9.1281 9734556

[B43] Honey G. Corlett P. Absalom A. Lee M. Pomarol-Clotet E. Murray G. (2008). Individual differences in psychotic effects of ketamine are predicted by brain function measured under placebo. *J. Neurosci.* 28 6295–6303. 10.1523/JNEUROSCI.0910-08.2008 18562599 PMC3838937

[B44] Hong L. Moran L. Du X. O’Donnell P. Summerfelt A. (2012). Mismatch negativity and low frequency oscillations in schizophrenia families. *Clin. Neurophysiol.* 123 1980–1988. 10.1016/j.clinph.2012.03.011 22541739 PMC3436985

[B45] Hsiao F. Cheng C. Liao K. Lin Y. (2010). Cortico-cortical phase synchrony in auditory mismatch processing. *Biol. Psychol.* 84 336–345. 10.1016/j.biopsycho.2010.03.019 20380866

[B46] Hsiao F. Wu L. Yung-Yang L. (2009). Theta oscillation during auditory change detection: an MEG study. *Biol. Psychol.* 81 58–66. 10.1016/j.biopsycho.2009.01.007 19428969

[B47] Hunt M. Kasicki S. (2013). A systematic review of the effects of NMDA receptor antagonists on oscillatory activity recorded in vivo. *J. Psychopharmacol.* 27 972–986. 10.1177/0269881113495117 23863924

[B48] Javitt D. (2000). Intercortical mechanisms of mismatch negativity dysfunction in schizophrenia. *Audiol. Neurootol.* 5 207–215. 10.1159/000013882 10859415

[B49] Javitt D. (2009a). Sensory processing in schizophrenia: neither simple nor intact. *Schizophr. Bull.* 35 1059–1064. 10.1093/schbul/sbp110 19833806 PMC2762632

[B50] Javitt D. (2009b). When doors of perception close: bottom-up models of disrupted cognition in schizophrenia. *Clin. Psychol.* 5 249–275. 10.1146/annurev.clinpsy.032408.153502 19327031 PMC4501390

[B51] Javitt D. (2015). Neurophysiological models for new treatment development in schizophrenia: early sensory approaches. *Ann. N. Y. Acad. Sci.* 1344 92–104. 10.1111/nyas.12689 25721890 PMC4467888

[B52] Javitt D. Freedman R. (2015). Sensory processing dysfunction in the personal experience and neuronal machinery of schizophrenia. *Am. J. Psychiatry* 172 17–31. 10.1176/appi.ajp.2014.13121691 25553496 PMC4501403

[B53] Javitt D. Lee M. Kantrowitz J. Martinez A. (2018). Mismatch negativity as a biomarker of theta band oscillatory dysfunction in schizophrenia. *Schizophr. Res.* 191 51–60. 10.1016/j.schres.2017.06.023 28666633

[B54] Javitt D. Spencer K. Thaker G. Winterer G. Hajós M. (2008). Neurophysiological biomarkers for drug development in schizophrenia. *Nat. Rev. Drug Discov.* 7 68–83. 10.1038/nrd2463 18064038 PMC2753449

[B55] Javitt D. Steinschneider M. Schroeder C. Arezzo J. (1996). Role of cortical N-methyl-D-aspartate receptors in auditory sensory memory and mismatch negativity generation: implications for schizophrenia. *Proc. Natl. Acad. Sci. U.S.A.* 93 11962–11967. 10.1073/pnas.93.21.11962 8876245 PMC38166

[B56] Javitt D. Sweet R. (2015). Auditory dysfunction in schizophrenia: integrating clinical and basic features. *Nat. Rev. Neurosci.* 16 535–550. 10.1038/nrn4002 26289573 PMC4692466

[B57] Javitt D. Zukin S. Heresco-Levy U. Umbricht D. (2012). Has an angel shown the way? Etiological and therapeutic amplifications of the PCP/NMDA model of schizophrenia. *Schizophr. Bull.* 38 958–966. 10.1093/schbul/sbs069 22987851 PMC3446214

[B58] Jobert M. Wilson F. Ruigt G. Brunovsky M. Prichep L. Drinkenburg W. (2012). Guidelines for the recording and evaluation of pharmaco-EEG data in man: the international pharmaco-EEG society (IPEG). *Neuropsychobiology* 66 201–220. 10.1159/000343478 23075830

[B59] Kantrowitz J. Epstein M. Beggel O. Rohrig S. Lehrfeld J. Revheim N. (2016). Neurophysiological mechanisms of cortical plasticity impairment in schizophrenia and modulation by the NMDA receptor agonist D-serine. *Brain* 139 3281–3295. 10.1093/brain/aww262 27913408 PMC5840885

[B60] Kantrowitz J. Epstein M. Lee M. Lehrfeld N. Nolan K. Shope C. (2018). Improvement in mismatch negativity generation during d-serine treatment in schizophrenia: correlation with symptoms. *Schizophr. Res.* 191 70–79. 10.1016/j.schres.2017.02.027 28318835

[B61] Kantrowitz J. Hoptman M. Leitman D. Moreno-Ortoga M. Lehrfeld N. Dias E. (2015). Neural substrates of auditory emotion recognition deficits in schizophrenia. *J. Neurosci.* 35 14909–14921. 10.1523/JNEUROSCI.4603-14.2015 26538659 PMC4635137

[B62] Kantrowitz J. Javitt D. (2010). N-methyl-D-aspartate (n.d.) receptor dysfunction or dysregulation: the final common pathway on the road to schizophrenia? *Brain Res. Bull.* 30 108–121. 10.1016/j.brainresbull.2010.04.006 20417696 PMC2941541

[B63] Kasai K. (2004). Structural and functional abnormalities of the auditory cortex in schizophrenia. *Int. Congr. Ser.* 1270 50–55. 10.1016/j.ics.2004.04.052

[B64] Kasai K. Nakagome K. Itoh K. Koshida I. Iwanami A. Fukuda M. (2002). Impaired cortical network for preattentive detection of change in speech sounds in schizophrenia: a high-resolution event-related potential study. *Am. J. Psychiatry* 159:4. 10.1176/appi.ajp.159.4.546 11925291

[B65] Kaser M. Soltesz F. Lawrence P. Miller S. Dodds C. Croft R. (2013). Oscillatory underpinnings of mismatch negativity and their relationship with cognitive function in patients with schizophrenia. *PLoS One* 8:e83255. 10.1371/journal.pone.0083255 24358266 PMC3866183

[B66] Kawakubo Y. Kamco S. Nose T. Iwanami A. Nakagome K. Fukuda M. (2007). Phonetic mismatch negativity predicts social skills acquisition in schizophrenia. *Psychiatry Res.* 152 261–265. 10.1016/j.psychres.2006.02.010 17521744

[B67] Kawakubo Y. Kasai K. Kudo N. Rogers M. Nakagome K. Itoh K. (2006). Phonetic mismatch negativity predicts verbal memory deficits in schizophrenia. *Neuroreport* 17 1043–1046. 10.1097/01.wnr.0000221828.10846.ba 16791100

[B68] Kawakubo Y. Suga M. Tochigi M. Yumoto M. Itoh K. Sasaki T. (2011). Effects of metabotropic glutamate receptor 3 genotype on phonetic mismatch negativity. *PLoS One* 6:e24929. 10.1371/journal.pone.0024929 22022368 PMC3191133

[B69] Kircher T. Rapp A. Grodd W. Buchkremer G. Weiskopf N. Lutzenburger W. (2004). Mismatch negativity response in schizophrenia: a combined fMRI and whole-head MEG study. *Am. J. Psychiatry* 161 294–304. 10.1176/appi.ajp.161.2.294 14754779

[B70] Kirino E. (2017). Mismatch negativity correlates with delta and theta EEG power in schizophrenia. *Int. J. Neurosci.* 17 1257–1279. 10.1080/00207450600936635 17654091

[B71] Knott V. (2000). Quantitative EEG methods and measures in human psychopharmacological research. *Hum. Psychopharmacol.* 15 479–498. 10.1002/1099-1077(200010)15:7<479::AID-HUP206<3.0.CO;2-5 12404618

[B72] Knott V. McIntosh J. Millar A. Fisher D. Villeneuve C. Ilivitsky V. (2006). Nicotine and smoker status moderate brain electric and mood activation induced by ketamine, and N-methyl-D-aspartate receptor antagonist. *Pharmacol. Biochem. Behav.* 85 228–242. 10.1016/j.pbb.2006.08.005 17023037

[B73] Ko D. Kwon S. Lee G. Im H. Kim H. Jung K. (2012). Theta oscillation related to the auditory discrimination process in mismatch negativity: oddball versus control paradigm. *J. Clin. Neurosci.* 8 35–42. 10.3988/jcn.2012.8.1.35 22523511 PMC3325430

[B74] Kocsis B. Brown R. McCarley R. Hajos M. (2013). Impact of ketamine on neuronal network dynamics: translational modelling of schizophrenia-relevant deficits. *CNS Neurosci. Ther.* 19 437–447. 10.1111/cns.12081 23611295 PMC3663928

[B75] Koerrner T. Zhang Y. Nelson P. Wang B. Zou N. (2016). Neural indices of phonemic discrimination and sentence-level intelligibility in quiet and noise: a mismatch negativity study. *Hear. Res.* 339 40–49. 10.1016/j.heares.2017.04.009 27267705

[B76] Kreitschmann-Andermahr I. Rosburg T. Mejer T. Volz H. Nowak H. Saver H. (1999). Impaired sensory processing in male patients with schizophrenia: a magnetoencephalographic study of auditory mismatch detection. *Schizophr. Res.* 35 121–129. 10.1016/s0920-9964(98)00115-7 9988849

[B77] Krystal J. D’Souza D. Karper L. Bennett A. Abi-Dargham A. Abi-Saad D. (1999). Interactive effects of subanesthetic ketamine and haloperidol in healthy humans. *Psychopharmacoloy (Berl.)* 45 193–204. 10.1007/s002130051049 10463321

[B78] Lahti A. Holcomb H. Medoff D. Tamminga C. (1995). Ketamine activates psychosis and alters limbic blood flow in schizophrenia. *Neuroreport* 6 869–872. 10.1097/00001756-199504190-00011 7612873

[B79] Lahti A. Weiler M. Michaelidis T. Parweni A. Tamminga C. (2001). Effects of ketamine in normal and schizophrenic volunteers. *Neuropsychopharmacoloy* 25 455–467. 10.1016/S0893-133X(01)00243-3 11557159

[B80] Lavoie S. Murray M. Deppen P. Knyazeva M. Berk M. Boulat O. (2008). Glutathione precursor, N-acetyl-cysteine, improves mismatch negativity in schizophrenia patients. *Neuropsychopharmacology* 33 2187–2199. 10.1038/sj.npp.1301624 18004285

[B81] Lazarewicz M. Ehrlichman R. Maxwell C. Gandal M. Finkel L. Siegel S. (2009). Ketamine modulates theta and gamma oscillations. *J. Cog. Neurosci.* 22 1452–1464. 10.1162/jocn.2009.21305 19583475

[B82] Lee M. Balla A. Sershen H. Sehatpour P. Lakatos P. Javitt D. (2018). Rodent mismatch negativity/theta neuro-oscillatory response as a translational neurophysiological biomarker for N-methyl-D-aspartate receptor-based new treatment development in schizophrenia. *Neuropsychopharmacology* 43 571–582. 10.1038/npp.2017.176 28816240 PMC5770758

[B83] Lee M. Sehatpour P. Hoptman M. Lakatos P. Dias E. Kantrowitz J. (2017). Neural mechanisms of mismatch negativity (MMN) dysfunction in schizophrenia. *Mol. Psychiatry* 22 1585–1593. 10.1038/mp.2017.3 28167837 PMC5547016

[B84] Leitman D. Laukka P. Joslin P. Saccente E. Butler P. Javitt D. (2010a). Getting the cue: sensory contributions to auditory emotion recognition impairments in schizophrenia. *Schizophr. Bull.* 36 545–556. 10.1093/schbul/sbn115 18791077 PMC2879690

[B85] Leitman D. Wolf D. Ragland J. Laukka P. Loughead J. Valdez J. (2010b). “It’s not what you say, but how you say it”: a reciprocal temporo-frontal network for affective prosody. *Front. Neurosci.* 4:19. 10.3389/fnhum.2010.00019 20204074 PMC2831710

[B86] Leitman D. Schatpour P. Higgins B. Foxe J. Silipo G. (2010). Sensory deficits and distributed hierarchical dysfunction in schizophrenia. *Am. J. Psychiatry* 167 818–827. 10.1176/appi.ajp.2010.09030338 20478875

[B87] Leung S. Croft R. O’Neill B. Nathan P. (2008). Acute high-dose glycine attenuates mismatch negativity (MMN) in healthy human controls. *Psychopharmacology* 196 451–460. 10.1007/s00213-007-0976-8 17952411

[B88] Malhotra A. Adler C. Kinnison S. Elman I. Pickar D. Breier A. (1997). Clozapine blunts N-methyl-D-aspartate antagonist-induced psychosis: a study with ketamine. *Biol. Psychiatry* 42 664–668. 10.1016/s0006-3223(96)00546-x 9325559

[B89] Maxwell M. (1992). *Family Interview for Genetic Studies (FIGS): Manual for FIGS. Clinical Neurogenetics Branch, Intramural Research Program*. Bethesda, MD: National Institute of Mental Health.

[B90] Miyanishi T. Sumiyoshi T. Higuchi Y. Seo T. Suzuki M. (2013). LORETA current source density for duration mismatch negativity and neuropsychological assessment in early schizophrenia. *PLoS One* 8:e61152. 10.1371/journal.pone.0061152 23577204 PMC3618448

[B91] Moghaddam R. Javitt D. (2012). From revolution to evolution: the glutamate hypothesis of schizophrenia and its implications for treatment. *Neuropsychopharmacology* 37 4–15. 10.1038/npp.2011.181 21956446 PMC3238069

[B92] Moghaddam R. Krystal J. (2012). Capturing the angel in “angel dust”: twenty years of translational neuroscience studies of NMDA receptor antagonists in animals and humans. *Schizophr. Bull.* 38 942–949. 10.1093/schbul/sbs075 22899397 PMC3446228

[B93] Moran L. Hong E. (2011). High and low frequency neural oscillations in schizophrenia. *Schizophr. Bull.* 37 659–663. 10.1093/schbul/sbr056 21653278 PMC3122299

[B94] Morgan C. Mofeez A. Brandner B. Bromley L. Curran H. (2004). Acute effects of ketamine on memory systems and psychotic symptoms in healthy volunteers. *Neuropsychopharmacology* 29 208–218. 10.1038/sj.npp.1300342 14603267

[B95] Morgan C. Perry E. Cho H. Krystal J. D’Souza D. (2006). Greater vulnerability to the amnestic effects of ketamine in males. *Psychopharmacology* 187 405–414. 10.1007/s00213-006-0409-0 16896964

[B96] Näätänen R. (1999). Phoneme representations of the human brain as reflected by event-related potentials. *Electroencephalogr. Clin. Neurophysiol. Suppl.* 49 170–173. 10533104

[B97] Näätänen R. Alho K. (1997). Mismatch negativity – The measure for central sound representation accuracy. *Audiol. Neurootol.* 2 341–353. 10.1159/000259255 9390839

[B98] Näätänen R. Kakkonen S. (2009). Central auditory dysfunction in schizophrenia as revealed by the mismatch negativity (MMN) and its magnetic equivalent MMNs: a review. *Int. J. Neuropsychopharmacol.* 12 125–135. 10.1017/S1461145708009322 18771603

[B99] Näätänen R. Paavilainen P. Rinne T. Alho K. (2007). The mismatch negativity (MMN) is basic research of central auditory processing: a review. *Clin. Neurophysiol.* 118 2544–2590. 10.1016/j.clinph.2007.04.026 17931964

[B100] Näätänen R. Pakarinen S. Rinne T. Takegata R. (2004). The mismatch negativity (MMN)- towards the optimal paradigm. *Clin. Neurophysiol.* 115 140–144. 10.1016/j.clinph.2003.04.001 14706481

[B101] Näätänen R. Todd J. Schall U. (2015). Mismatch negativity (MMN) as biomarker predicting psychosis in clinically at-risk individuals. *Biol. Psychiatry* 116 36–40. 10.1016/j.biopsycho.2015.10.010 26542526

[B102] Northoff G. (2014). Are auditory hallucinations related to the brain’s resting state activity? A “neurophenomenal resting state” hypothesis. *Clin. Psychopharmacol. Neurosci.* 12 189–195. 10.9758/cpn.2014.12.3.189 25598821 PMC4293163

[B103] Northoff G. (2015). Resting state activity and the “stream of consciousness” in schizophrenia – Neurophenomenal hypotheses. *Schizophr. Bull.* 41 280–290. 10.1093/schbul/sbu116 25150784 PMC4266297

[B104] Northoff G. Qin P. (2011). How can the brain’s resting state activity generate hallucinations? A “resting state hypothesis” of auditory verbal hallucinations. *Schizophr. Res.* 127 202–214. 10.1016/j.schres.2010.11.009 21146961

[B105] Northoff G. Richter A. Bermpohl F. Grimm S. Martin V. (2005). NMDA hypofunction in the posterior cingulate as a model for schizophrenia: an exploratory ketamine administration study in fMRI. *Schizophr. Res.* 72 235–248. 10.1016/j.schres.2004.04.009 15560968

[B106] Opitz B. Rinne T. Mecklinger A. von Cramon D. Schroger E. (2002). Differential contribution of frontal and temporal cortices to auditory change detection: fMRI and ERP results. *Neuroimage* 15 167–174. 10.1006/nimg.2001.0970 11771985

[B107] Paavilainen P. (2013). The mismatch-negativity (MMN) component of the auditory event-related potential to violations of abstract regularities: a review. *Int. J. Psychophysiol.* 88 109–123. 10.1016/j.ijpsycho.2013.03.015 23542165

[B108] Pakarinen S. Lovio R. Huotilaninen M. Alku P. Näätänen R. Kujala T. (2009). Fast multi-feature paradigm for recording several mismatch negativities (MMNs) to phonetic and acoustic changes in speech sounds. *Biol. Psychol.* 82 219–226. 10.1016/j.biopsycho.2009.07.008 19646504

[B109] Park H. Kwon J. Youn T. Pae J. Kim J. Kim M. (2002). Statistical parametric mapping of LORETA using high density EEG and individual MRI: application to mismatch negativity in schizophrenia. *Hum. Brain Mapping.* 17 168–178. 10.1002/hbm.10059 12391570 PMC6872044

[B110] Pascual-Marqui R. (2007). Discrete, 3D distributed, linear imaging methods of electric neuronal activity. Part 1: exact, zero error localization. arXiv:0710.3341 [Preprint].

[B111] Pascual-Marqui R. Lehman D. Koukkou M. Kochi K. Anderer P. Saletu B. (2011). Assessing interactions in the brain with exact low resolution electromagnetic tomography (eLORETA). *Philos. Trans. A Math. Phys. Eng.* 360 3768–3784. 10.1098/rsta.2011.0081 21893527

[B112] Pauvermann M. Lee G. Dawson M. (2017). Glutamatergic regulation of cognition and functional brain connectivity: insights from pharmacological, genetic and translational schizophrenia research. *Br. J. Psychiatry* 174 3136–3160. 10.1111/bph.13919 28626937 PMC5595770

[B113] Pekkonen E. Katila H. Ahveninen J. Karhu J. Hootilainen M. Tiihonen J. (2002). Impaired temporal lobe processing of preattentive auditory discrimination in schizophrenia. *Schizophr. Bull.* 28 467–474. 10.1093/oxfordjournals.schbul.a006954 12645678

[B114] Pulvermuller F. Shtyrov Y. (2006). Language outside the focus of attention: the mismatch negativity as a tool for studying higher cognitive processes. *Prog. Neurobiol.* 79 49–71. 10.1016/j.pneurobio.2006.04.004 16814448

[B115] Retsa C. Knebel J. Geiser E. Ferrari C. Jenni R. Fournier M. (2018). Treatment in early psychosis with N-acetyl-cysteine for 6 months improves low-level auditory processing: a pilot study. *Schizophr. Res.* 191 80–86. 10.1016/j.schres.2017.07.008 28711476

[B116] Revheim N. Corcoran C. Dias E. Hellmann E. Martinez A. Butler P. (2014). Reading deficits in schizophrenia and individuals at high clinical risk: relation to sensory function, course of illness, and psychosocial outcome. *Am. J. Psychiatry* 171 949–959. 10.1176/appi.ajp.2014.13091196 25178752 PMC4501394

[B117] Rodionov V. Durst R. Mager M. Teitelbaum A. Rashin S. (2009). Wavelet analysis of the frontal auditory evoked potentials obtained in the passive oddball paradigm in healthy subjects and schizophrenics. *J. Basic Clin. Physiol. Pharmacol.* 20 233–264. 19852310 10.1515/jbcpp.2009.20.3.233

[B118] Rosburg T. Kreitschmann-Andermahr H. (2016). The effects of ketamine on the mismatch negativity (MMN) in humans – A meta-analysis. *Clin. Neurophysiol.* 127 1387–1394. 10.1016/j.clinph.2015.10.062 26699665

[B119] Saletu B. Anderer P. Saletu-Zyblarz G. (2006). EEG topography and tomography (LORETA) in the classification and evaluation of the pharmacodynamics of psychotropic drugs. *Clin. EEG Neurosci.* 37 66–80. 10.1177/155005940603700205 16733939

[B120] Salisbury D. McCathern A. Coffman B. Murphy T. Haigh S. (2018). Complex mismatch negativity to tone pair deviants in long-term schizophrenia and in the first-episode schizophrenia spectrum. *Schizophr. Res.* 191 18–24. 10.1016/j.schres.2017.04.044 28506707 PMC5768305

[B121] Schall U. Muller B. Kargel C. Gunturkun O. (2015). Electrophysiological mismatch response recorded in awake pigeons from the avian functional equivalent of the primary auditory cortex. *Neuroreport* 26 239–244. 10.1097/WNR.0000000000000323 25646582

[B122] Schuelert N. Dorner-Ciosse C. Brendel M. Rosenbrock H. (2018). A comprehensive analysis of auditory event-related potentials and network oscillations in an NMDA receptor antagonist mouse model using a novel wireless recording technology. *Physiol. Rep.* 6:e13782. 10.14814/phy2.13782 30155997 PMC6113138

[B123] Shtyrov Y. Pulvermuller F. (2007). Language in the mismatch negativity design: motivations, benefits and prospects. *J. Psychophysiol.* 21 176–187. 10.1027/0269-8803.21.34.176

[B124] Steullet P. Neijt H. Cuenod M. Do K. (2006). Synaptic plasticity impairment and hypofunction of NMDA recptors induced by glutathione deficit: relevance to schizophrenia. *Neuroscience* 137 807–819. 10.1016/j.neuroscience.2005.10.014 16330153

[B125] Stone J. Erlandsson K. Arstad E. Squassante L. Tenneggi V. Bressan R. (2008). Relationship between ketamine-induced psychotic symptoms and NMDA receptor occupancy: a [(123)I]CNS-1261 SPET study. *Psychopharmacology* 197 401–408. 10.1007/s00213-007-1047-x 18176855

[B126] Takahashi H. Rissling A. Pascual-Marqui R. Kirihara K. Pela M. Sprock J. (2013). Neural substrates of normal and impaired preattentive sensory discrimination in large cohorts of nonpsychiatric subjects and schizophrenia patients as indexed by MMN and P3a detection responses. *Neuroimage* 1 594–603. 10.1016/j.neuroimage.2012.09.074 23085112 PMC3652903

[B127] Tallon-Baudry C. Bertrand O. Delpuech C. Pernier J. (1996). Stimulus specificity of phase-locked and non-phase-locked 40 Hz visual responses in human. *J. Neurosci.* 16 4240–4249. 10.1523/jneurosci.16-13-04240.1996 8753885 PMC6579008

[B128] Tamminen H. Peltola M. Kujala T. Naatanen R. (2015). Phonetic training and non-native speech perception – How memory traces evolve in just three days as indexed by the mismatch negativity (MMN) and behavioural measures. *Int. J. Psychophysiol.* 97 23–29. 10.1016/j.ijpsycho.2015.04.020 25956191

[B129] Thiebes S. Leicht G. Curic S. Steinmann S. Polomac N. Andreou C. (2017). Glutamatergic deficits and schizophrenia-like negative symptoms: new evidence from ketamine-induced mismatch negativity alternations in healthy male humans. *J. Psychiatry Neurosci.* 42 273–283. 10.2174/1389201019666180620112528 28556775 PMC5487274

[B130] Thomas E. Bozaoglu K. Russell S. Gurvich C. (2017). The influence of the glutamatergic system in cognition in schizophrenia: a systematic review. *Neurosci. Biobehav. Rev.* 77 369–397. 10.1016/j.neubiorev.2017.04.005 28414078

[B131] Thonnesen H. Zvyagintsev M. Harke K. Boers F. Dammers J. Norra C. (2008). Optimized mismatch negativity paradigm reflects deficits in schizophrenia patients. *Biol. Psychol.* 17 205–216. 10.1016/j.biopsycho.2007.10.009 18060677

[B132] Tikhonravov D. Neuvonen T. Pertovaara A. Savioja K. Ruusuvirta T. Näätänen R. (2008). Effects of an NMDA-receptor antagonist MK-801 on an MMN-like response recorded in anaesthetized rats. *Brain. Res.* 1203 97–102. 10.1016/j.brainres.2008.02.006 18325485

[B133] Todd J. Harms L. Schall U. Michi P. (2013). Mismatch negativity: translating the potential. *Front. Psychiatry* 4:171. 10.3389/fpsyt.2013.00171 24391602 PMC3866657

[B134] Todd J. Michie P. Jablensky A. (2003). Association between reduced duration mismatch negativity (MMN) and raised temporal discrimination thresholds in schizophrenia. *Clin. Neurophysiol.* 114 2061–2070. 10.1016/s1388-2457(03)00246-3 14580604

[B135] Todd J. Michie P. Schall U. Karayansdis F. Yabe H. Naatanen R. (2008). Deviant matters: duration, frequency, and intensity deviants reveal different patterns of mismatch negativity reduction in early and late schizophrenia. *Biol. Psychiatry* 63 58–64. 10.1016/j.biopsych.2007.02.016 17585889

[B136] Todd J. Michie P. Schall U. Ward P. Catts S. (2012). Mismatch negativity (MMN) reduction in schizophrenia – Impaired prediction-error generation, estimation or salience. *Int. J. Psychophysiol.* 83 222–231. 10.1016/j.ijpsycho.2011.10.003 22020271

[B137] Tramo M. Shah G. Braida L. (2002). Functional role of auditory cortex in frequency processing and pitch perception. *J. Neurophysiol.* 87 122–139. 10.1152/jn.00104.1999 11784735

[B138] Tse C. Rinne T. Ng K. Penney T. (2013). The functional role of the frontal cortex in pre-attentive auditory change detection. *Neuroimage* 83 870–879. 10.1016/j.neuroimage.2013.07.037 23871868

[B139] Umbricht D. Koller R. Vollenweider F. Schmid L. (2002). Mismatch negativity predicts psychotic experiences induced by NMDA receptor antagonist in healthy volunteers. *Biol. Psychiatry* 51 400–406. 10.1016/s0006-3223(01)01242-2 11904134

[B140] Umbricht D. Vyssotki D. Latanov A. Nitsch R. Lipp H. (2005). Deviance-related electrophysiological activity in mice: is there mismatch negativity in mice? *Clin. Neurophysiol.* 116 353–363. 10.1016/j.clinph.2004.08.015 15661113

[B141] Vollenweider F. Lienders K. Scharfetter C. Antagonini A. Maguire P. Missimer J. (1987). Metabolic hyperfrontality and psychopathology in the ketamine model of psychosis using positron emission tomography (PET) and [18F] flourodeoxyglucose (FDG). *Eur. Neuropsychopharmacology* 7 9–24. 10.1016/s0924-977x(96)00039-9 9088881

[B142] Wang Z. Ryan A. Woolf N. (1987). Pentobarbital and ketamine alter the patters of 2-deoxyglucose uptake in the central auditory system of the gerbil. *Hear. Res.* 27 145–155. 10.1016/0378-5955(87)90015-3 3610843

[B143] Wible C. Kobicki M. Yoo S. Kacher D. Jalisbury D. Anderson M. (2001). A functional magnetic resonance imaging study of auditory mismatch negativity in schizophrenia. *Am. J. Psychiatry* 158 938–943. 10.1176/appi.ajp.158.6.938 11384903 PMC2845157

[B144] Williams J. Gibbon M. First M. Spitzer R. Davies M. Borus J. (1992). The structured Clinical Interview for the DSM-III-R (SCID). Multisite test-retest reliability. *Arch. Gen. Psychiatry* 49 630–636. 1637253 10.1001/archpsyc.1992.01820080038006

[B145] Womelsdorf T. Valiante T. Sahin N. Miller K. Tiesinga P. (2014). Dynamic circuit motifs underlying rhythmic gain control, gating and integration. *Nat. Neurosci.* 17 1031–1039. 10.1038/nn.3764 25065440

[B146] Xiong Y. Bo Q. Wang C. Tian Q. Liu Y. Wang C. (2019). Differential of frequency and duration mismatch negativity and theta power deficits in first-episode and chronic schizophrenia. *Front. Behav. Neurosci.* 6:37. 10.3389/fnbeh.2019.00037 30894804 PMC6414796

[B147] Yamasue H. Yamada H. Yumoto M. Kamco S. Kudo N. Vetsuki M. (2004). Abnormal association between reduced magnetic mismatch field to speech sounds and smaller planum temporale volume in schizophrenia. *Neuroimage* 22 720–727. 10.1016/j.neuroimage.2004.01.042 15193600

[B148] Youn T. Park H. Kim J. Kim M. Kwon J. (2003). Altered hemispheric symmetry and positive symptoms in schizophrenia: equivalent current dipole of auditory mismatch negativity. *Schizophr. Res.* 59 253–260. 10.1016/s0920-9964(02)00154-8 12414082

